# The “ram effect”: new insights into neural modulation of the gonadotropic axis by male odors and socio-sexual interactions

**DOI:** 10.3389/fnins.2015.00111

**Published:** 2015-04-09

**Authors:** Claude Fabre-Nys, Keith M. Kendrick, Rex J. Scaramuzzi

**Affiliations:** ^1^UMR 7247 Physiologie de la Reproduction et des Comportements, Centre National de la Recherche Scientifique, Institut National de la Recherche Agronomique, Institut Français du Cheval et de L'équitation, Université de ToursNouzilly, France; ^2^Key Laboratory for Neuroinformation, University of Electronic Science and Technology of ChinaChengdu, China; ^3^Department of Comparative Biological Sciences, Royal Veterinary CollegeSouth Mimms, UK

**Keywords:** ram effect, odor, LH, estradiol, main olfactory system, experience, noradrenaline, kisspeptin

## Abstract

Reproduction in mammals is controlled by the hypothalamo-pituitary-gonadal (HPG) axis under the influence of external and internal factors such as photoperiod, stress, nutrition, and social interactions. Sheep are seasonal breeders and stop mating when day length is increasing (anestrus). However, interactions with a sexually active ram during this period can override the steroid negative feedback responsible for the anoestrus state, stimulate luteinizing hormone (LH) secretion and eventually reinstate cyclicity. This is known as the “ram effect” and research into the mechanisms underlying it is shedding new light on HPG axis regulation. The first step in the ram effect is increased LH pulsatile secretion in anestrus ewes exposed to a sexually active male or only to its fleece, the latter finding indicating a “pheromone-like” effect. Estradiol secretion increases in all ewes and this eventually induces a LH surge and ovulation, just as during the breeding season. An exception is a minority of ewes that exhibit a precocious LH surge (within 4 h) with no prior increase in estradiol. The main olfactory system and the cortical nucleus of the amygdala are critical brain structures in mediating the ram effect since it is blocked by their inactivation. Sexual experience is also important since activation (increased c-fos expression) in these and other regions is greatly reduced in sexually naïve ewes. In adult ewes kisspeptin neurons in both arcuate and preoptic regions and some preoptic GnRH neurons are activated 2 h after exposure to a ram. Exposure to rams also activates noradrenergic neurons in the locus coeruleus and A1 nucleus and increased noradrenalin release occurs in the posterior preoptic area. Pharmacological modulation of this system modifies LH secretion in response to the male or his odor. Together these results show that the ram effect can be a fruitful model to promote both a better understanding of the neural and hormonal regulation of the HPG axis in general and also the specific mechanisms by which male cues can overcome negative steroid feedback and trigger LH release and ovulatory cycles.

## Introduction

Reproduction is essential for the survival and evolution of species and in most vertebrates it is controlled by similar networks of hormonal signals. The key regulator of the network is the hypothalamic neuropeptide, gonadotrophin releasing hormone (GnRH), which controls the release of the pituitary hormones, luteinizing hormone (LH), and follicle stimulating hormone (FSH). These latter hormones then stimulate the gonads to produce functional gametes and secrete estradiol, progesterone and testosterone that sustain reproductive function and auto-regulate the activity of the gonadotrophic axis by modulating the secretion of GnRH, LH, and FSH through positive and negative feedback systems. The mechanisms involved in the regulation of this network, often referred to as the hypothalamo-pituitary gonadal axis (HPG), have been the object of abundant research for several decades (Knobil, 1981; Karsch, 1984) but a central question which remained unresolved was how sex steroids modulate the activity of GnRH neurons while the latter lack receptors for steroids involved in feedback action (Herbison, 1998). However, the discovery of kisspeptin-containing neurons as being the most potent secretagogues of GnRH (Messager et al., 2005), and the recent observation that, in mice, all kisspeptin neurons projecting on GnRH neurons have estradiol receptors (Kumar et al., 2015) make them the most probable target of steroid action (Clarkson and Herbison, 2009) and has opened up a new era in this field of research.

The HPG axis is also modulated by many internal and external factors such as nutrition, stress, immunological status, physical, and social environment (Signoret, 1980; Tomaszewska-Zaremba and Herman, 2009; Dobson et al., 2012; Follett, 2014; Roa and Tena-Sempere, 2014) but the mechanisms involved are largely unclear. The effects of the social environment are particularly intriguing because they are very diverse. In mammals they can inhibit reproduction such as in naked mole rat social groups in which reproduction is restricted to a few individuals (Goldman et al., 2006). This is also the case in marmoset family groups where the presence of the mother inhibits reproduction of her daughters (Abbott et al., 1981) or in mice where overcrowding can block reproduction (Whitten, 1959). In contrast, the presence of a sexual partner in many mammalian species can stimulate reproduction (Signoret, 1980; Vandenbergh, 2006) and may even be necessary for females to ovulate, such as in the cat, rabbit, and camel (Bakker and Baum, 2000). In sheep contact with a sexual partner has profound effects on reproductive events at all stages of reproductive life; it hastens puberty (Dyrmundsson and Lees, 1972), induces ovulation during seasonal anestrus (see review by Ungerfeld, 2007a,b) or lactational anestrus (Mauléon and Dauzier, 1965), and modifies the timing of the LH surge during the breeding season (Lindsay et al., 1975). The most spectacular and best known effect is the induction of ovulation in sexually quiescent females by exposure to a sexually active male, a phenomenon known as the “ram effect” in sheep (Martin et al., 1986; Ungerfeld, 2007a). This effect of a male has also been described in goats (Chemineau, 1983; Walkden-Brown et al., 1999) and in several wild ungulates (Skinner et al., 2002; Shipka et al., 2002). The “ram effect” was discovered in 1944 (Underwood et al., 1944) but studies on the mechanisms involved only started in the 80's when reliable LH assays became readily available and when the sheep, because of their size, availability and economic importance became a widely used model for the study of the HPG axis (Karsch et al., 1997). The object of this review is to summarize what we have learned in the last few decades about the mechanisms involved in the “ram effect” and to discuss how this knowledge could help us to understand the regulation of the HPG axis more generally.

## Description of the “ram effect”

**Figure 1:**
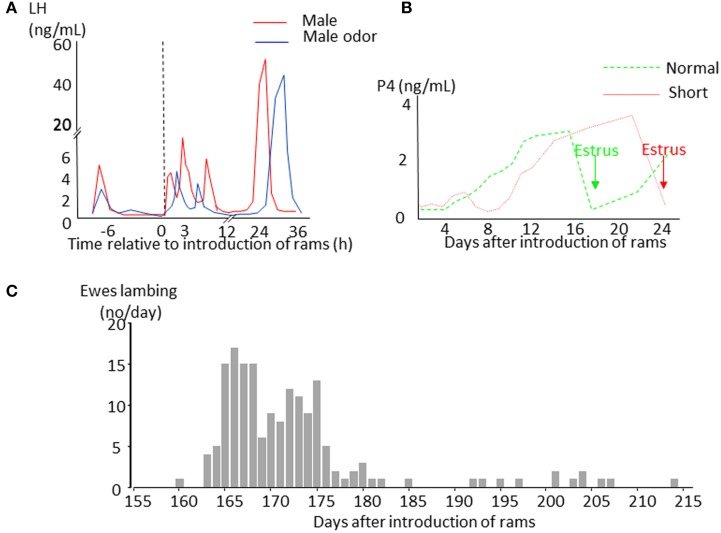
**Schematic representation of the effects of the introduction of sexually active rams to anestrus ewes**. **(A)** Changes in LH plasma concentrations in ewes exposed to a sexually active ram (red line) or to its fleece (blue line). **(B)** Changes in progesterone plasma concentrations indicating formation of a corpus luteum. In ewes presenting a luteal phase lasting around 10 days (normal cycle green line), estrus (green arrow) is displayed 16–19 days after male introduction before the second ovulation. In ewes with a short luteal phase (short cycle red line), estrus (red arrow) appears 21–26 days after ram introduction before the third ovulation. **(C)** Example of distribution of lambing in a flock of anestrus Mérinos d'Arles ewes exposed to the ram during the middle of anestrus. The ewes lambing during the first wave around 165 days after male introduction are those that first presented a normal cycle, those lambing in the second wave are those that first presented a short cycle. The ewes lambing later than 180 days are generally ones that did not became pregnant at their first mating.

Sheep are seasonal breeders. Ewes have regular 17 days oestrous cycles when day length is decreasing (the breeding season) and give birth in the spring when the environmental conditions are most favorable for the survival of their young. As day length increases ewes stop cycling (anestrus) but the introduction of a sexually active ram into a group of seasonally anoestrous animals will induce a pulse of LH (the short-term LH response Figure 1A) within minutes, whereas FSH does not undergo such a rapid change (Martin et al., 1980; Poindron et al., 1980). Exposure to a ram, or his odor, does not induce marked behavioral changes in ewes, but seems to focus their attention and induce urination (Gelez et al., 2004a). If contact with the male is maintained, the increase in pulsatile LH secretion initiates a sequence of physiological events that in some ewes will culminate in a LH surge 6–54 h later (Oldham et al., 1978; Chanvallon et al., 2011). Ovulation nearly always follows the LH surge (100% cases in Chanvallon et al., 2011; 97% in Scaramuzzi et al., 2014) but is described as “silent” because it is not associated with estrous behavior. Some females will then have a normal estrous cycle with a luteal phase lasting approximately 10 days and display estrous behavior 17–20 days after the introduction of rams (Figure 1B). In others, the corpus luteum from this first “silent” ovulation does not develop normally and regresses after a few days with a resultant short cycle; the ewe starts a new cycle but without a display of estrus (Oldham and Martin, 1978). Sexual behavior in these females only appears 22–28 days after the introduction of ram at the time of the third ovulation (Figure 1B). If ewes are mated at the time of the male induced estrus, a synchronized pattern of lambing occurs with two peaks 164 and 172 days afterwards. This singular pattern of births (Figure 1C) is the origin of the discovery of this phenomenon (Underwood et al., 1944) and could be used a convenient tool to identify those breeds responding to the “ram effect.”

Nearly all adult ewes have a short-term LH response after exposure to a sexually active ram during anestrus (93% in Chanvallon et al., 2011; 92% in Scaramuzzi et al., 2014). However, the intensity of this short term response varies and LH pulsatility after the “ram effect” is lower in ewes with low as opposed to high body condition (Scaramuzzi et al., 2014). An analysis of the LH response to a bolus of GnRH (75 ng) given to the same animals the day before they were exposed to the “ram effect” indicated that at least part of this variability was due to altered sensitivity of the pituitary. This was due to ewes with a low body condition having LH pulses of a lower amplitude in response to the GnRH bolus than those with a high body condition (Scaramuzzi et al., 2014).

A short-term LH response to the presence of a ram is not restricted to the anestrus period and is observed in some cycling ewes during the luteal phase (Hawken et al., 2007; Chanvallon et al., 2010a) although less frequently than in anestrous ones. This is rather surprising because progesterone is known to have a strong inhibitory action on LH secretion (Goodman and Karsch, 1980; Goodman et al., 2002) and suggests that the network by which male cues stimulate LH secretion is at least partially different from that involved in ovarian steroid feedback. Interestingly the intensity of short-term LH secretion is a parameter that can predict the occurrence of ovulation since LH pulse frequency after the introduction of rams is higher in ewes that subsequently ovulate than in ones that do not (Chanvallon et al., 2011).

In contrast to the high incidence of short-term LH responses to cues from the ram, the frequency of actual LH surges and resultant ovulations is much more variable ranging from 0 to 100%. This is dependent upon many factors, but especially on the breed, age, experience, nutritional state of the animals and time of the year (Oldham et al., 1978; Chanvallon et al., 2010a,b, 2011; Johnson et al., 2011), suggesting that the induction of the LH surge is the major cause of variability in response to the “ram effect.”

## Role of estradiol

**Figure 2:**
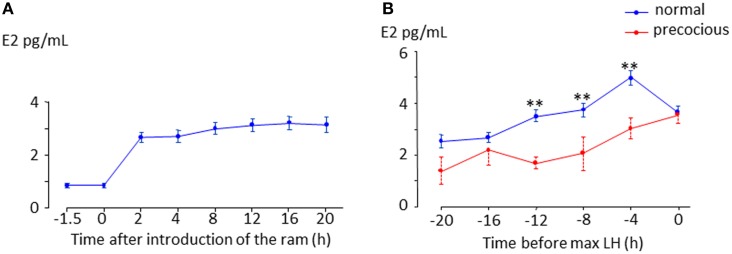
**Changes in plasma in anestrus ewes following the introduction of a ram**. **(A)** Data are means ± SEM of estradiol concentrations in 67 ewes of different breeds (Ile de France, Mérinos d'Arles, Mouton Vendéens and Romane) from the study by Chanvallon et al. (2011) after ram introduction at time 0. **(B)** Means ± SEM estradiol concentrations in the hours preceding a male induced-LH surge; blue line = ewes presenting a surge 8–56 h after male introduction (normal, *n* = 44 ewes of the four breeds above), dotted red line = ewes presenting a LH surge within 4 h after male introduction (precocious, *n* = 19 Ile de France and Mérinos d'Arles ewes). ^**^ indicate significant within time differences between the two groups at *p* < 0.001.

In sheep, as in all mammalian species, the LH surge is stimulated by an increase in secretion of the hypothalamic neuropeptide, GnRH induced by an increase in circulating estradiol (Hauger et al., 1977; Karsch et al., 1979; Goodman, 1994) during the follicular phase. This phenomenon referred to as “estradiol positive feedback,” lasts 12–24 h depending on the breed (Land et al., 1976; Cahill et al., 1981; Ben Saïd et al., 2007). It is widely assumed that the LH surge following the “ram effect” is induced by the same “estradiol positive feedback” mechanism (Martin et al., 1986). However, partly because of the difficulty in measuring the very low concentrations of circulating estradiol present during anestrus there has been very little experimental support for this hypothesis (Knight et al., 1978; Johnson et al., 2011). In a recent study we showed that, in all ewes the introduction of rams is followed by an increase in the circulating concentration of estradiol (Figure 2A, Fabre-Nys et al., 2015). In most anestrus ewes, the LH surge induced by the “ram effect” was preceded by increases in the circulating concentration of estradiol at least three-fold above the basal concentration for 14.5 ± 0.86 h (min 6 h; max 36 h, Figure 2B). Similar to the breeding season, the concentration of estradiol decreased at the time of the LH surge (Figure 2B).

The duration and pattern of these increases in estradiol concentrations varies among breeds. This variability in a highly seasonal breed, the Mouton Vendéen, is due to the low sensitivity of the ovary that releases very little amount of steroids in response to stimulation by a ram. The granulosa cells of these ewes in culture, also have a low response to *in vitro* stimulation by IGF-I and FSH and reduced expression of StAR (Fabre-Nys et al., 2015). In other breeds such as the Romane the frequency and latency of the LH surge is variable, although the quantities of estradiol secreted after the “ram effect” do not differ from those in breeds such as the Mérinos d'Arles and Ile de France that respond well to it. In these breeds the variability in response seems to be due to fluctuating sensitivity of the hypothalamo-hypophyseal complex to estradiol feedback.

In some ewes LH surges are induced immediately after exposure to a ram and these “precocious” LH surges are not preceded by increased concentrations of estradiol (Fabre-Nys et al., 2013; Figure 2B). Contrary to the spontaneous LH surges that occur in ewes during the breeding season, these “precocious” LH surges cannot be the result of classical “estradiol positive feedback.” Indeed the important question raised here is whether the mechanism of induction of these male-induced LH surges shares any similarities with that in spontaneous ovulators (Fabre-Nys et al., 2013).

## The stimuli involved

The ram emits a considerable range of different sensory stimuli that could be responsible for evoking a reproductive neuroendocrine response in ewes, but olfactory stimuli clearly play a dominant role. Direct physical contact with a ram is not necessary (Watson and Radford, 1960) and a complete reproductive neuroendocrine response can be induced by exposure to ram fleece (i.e., ram odor) alone (Knight and Lynch, 1980). However, fewer ewes ovulate when exposed only to ram odor (Pearce and Oldham, 1988) and additionally the frequency of short-term LH pulses is lower and they appear later (Gelez et al., 2004a). The active compounds involved are present in the fleece from all parts of the coat and in the anteorbital glands but are absent from urine (Cohen-Tannoudji et al., 1994). They are also androgen-dependant since ewes do not ovulate when exposed to a castrated ram (Fulkerson et al., 1981). A mixture of compounds is clearly involved because the biological activity of fleece requires the combined extracts of both the neutral and acidic fractions (Knight and Lynch, 1980; Cohen-Tannoudji et al., 1994). Those from the neutral fraction have been identified as 1,2-hexanedecanediol and 1,2-octanedecanediol, but those of the acidic fraction have not yet been identified (Cohen-Tannoudji et al., 1994).

The olfactory stimuli which generate the “ram effect” are not strictly species specific since hair from male goats stimulates LH pulsatile secretion (Over et al., 1990) and induces ovulation in ewes (Birch et al., 1989). A recent study showed that an acidic fraction of male goat hair that stimulated multiunit activity of the mediobasal hypothalamus of ovariectomized Shiba goats also stimulated pulsatile LH secretion in St Croix ewes (Ohara et al., 2014). The same group has also used this approach to show that 4-ethyloctanol is of key importance for activating the GnRH pulse generator in goats (Murata et al., 2014). This could therefore represent a very interesting way to approach the chemical identification of all the odorant compounds responsible for influencing the HPG axis.

However, the complete identification of all the active components of ram odor, might not be so simple a task because it appears that ewes need to “learn” to recognize them (see section below) and they can be trained to show an LH response to other odors. For example, ewes can show an increase in LH pulsatility in response to the odor of lavender if it has been associated with rams (Gelez et al., 2004a). This means that some odorant compounds may be common to both rams and bucks, some common to all rams but others may be specific to individual rams. Sheep can distinguish between odor cues from different individuals (Baldwin and Meese, 1977) and the presence of such individual olfactory signatures is important for mate selection in rodents (Brennan and Kendrick, 2006). In sheep it may help ewes identify specific rams and explain the increased response to newly introduced, “novel” ones (Jorre de St. Jorre et al., 2012).

Non-olfactory stimuli are also involved since short-term LH responses can be observed in anosmic ewes exposed to sexually active rams (Cohen-Tannoudji et al., 1986) indicating that other sensory inputs can substitute for male odor. The intensity of male sexual behavior is also important with males exhibiting high libido appearing to be more effective than males with low libido in inducing ovulation in some studies (Signoret et al., 1982; Perkins and Fitzgerald, 1994), although not in others (Fisher et al., 1994).Visual cues alone from rams alone have very limited effects with exposure of ewes to projected images of rams only inducing small increases in LH secretion (Hawken et al., 2009a). Auditory cues also seem to have very limited effects (Hawken et al., 2009a) which is not that surprising because not all rams vocalize when courting ewes.

## Factors affecting female sensitivity to male stimuli

### Breed and time of anestrus

The proportion of ewes ovulating in response to the “ram effect” varies with breed and time of year, the latter being highest in late anestrus (Ungerfeld, 2007b; Chanvallon et al., 2011). Sheep breeds also vary greatly in their sensitivity to photoperiodic cues (Malpaux, 2006).

Breeds are considered less seasonal if a high proportion of females are spontaneously cyclic during anestrus. This parameter is usually regarded as a sign of a “shallow” anestrus and is linked to a higher frequency of pulsatile LH secretion indicative of a lower response to the negative feedback of estradiol (Goodman et al., 1982). According to Lindsay and Signoret (1980) there is a positive correlation between the proportion of ewes in a flock that cycle spontaneously in anoestrus and that which ovulate after the “ram effect.” However, this theory has been challenged recently. In Limousine ewes, a moderately seasonal breed, Tournadre et al. (2002) found that the proportion of anestrus ewes ovulating was higher when there were fewer cyclic ewes in the flock. In another study comparing the responses of four French breeds of sheep Chanvallon et al. (2011) found that there was no link between the proportion of ewes ovulating after the “ram effect” and that of cyclic ewes present in the test flock. In some breeds, such as the Ile de France, ewes always show a high ovulatory response even if few females are cyclic in the flock, whereas the less seasonal Romane breed has highly variable ovulatory responses at the beginning of anestrus. This suggests the existence of a “sensitivity to socio-sexual stimulation” factor that in some conditions can override sensitivity to estradiol negative feedback.

### Experience and age

Young and sexually naïve ewes have a generally poorer ovarian response to the “ram effect” than adult, experienced ones (Oldham et al., 1984; Thimonier et al., 2000; Chanvallon et al., 2010a) even though they show good short-term LH responses (Gelez et al., 2004a). Pre-exposure of young ewes to rams several months before the “ram effect” increased the proportion of ewes ovulating in one study (Murtagh et al., 1984) but failed to do so in another (Chanvallon et al., 2010b), and had no effect on the short-term LH response (Gelez et al., 2004a). Pre-exposure to rams however increased the short-term LH response when the stimulus at the time of the ram effect was the odor of the fleece of the male (Gelez et al., 2004a). This effect involved some “learning” of male characteristics. If during the pre-exposure, the male had been scented with lavender some ewes showed a short-term LH response to lavender, whereas ewes exposed to an unscented ram or to unfamiliar ewes scented with lavender did not (Gelez et al., 2004a). However, the conditions required for the ewes to learn the necessary male characteristics and the mechanisms involved are currently unclear (Chanvallon et al., 2010b). They are likely to occur at the level of brain areas known to undergo structural changes associated with olfactory learning involved in social recognition such as the hippocampus, amygdala and olfactory bulb (Brennan and Keverne, 1997; Sanchez-Andrade and Kendrick, 2009). Additionally, recent studies have reported plasticity changes in pituitary gonadotrope cells with increases in cell numbers and connectivity being found after puberty and lactation (Budry et al., 2011; Alim et al., 2012; Hodson et al., 2012). Thus plasticity changes in the pituitary itself might also contribute to the effect of sexual experience on the response to the “ram effect.” Clearly more research is needed to improve our understanding of these conditions and to provide more information on the circuits that enhance the ability of the HPG axis to respond to environmental cues. This would also provide information of potential economic value to farmers wanting to improve the responses of their flocks to the “ram effect.”

### Stress

Stress can affect reproduction in many different ways (Rivier and Rivest, 1991; Ferin, 1993; Dobson et al., 2012). The abrupt change in the socio-sexual environment at the time of the “ram effect may be stressful and especially so for young sexually naïve ewes. However, this area of research has received very little attention. To examine the potential role of socio-sexual stress on modifying responses to the “ram effect” both adult sexually experienced and young sexually naïve Merino ewes genetically selected for “calm” or “nervous” temperaments over 15 generations (Murphy et al., 1994) were compared after “ram effect” (Chanvallon et al., 2010a). The hypothesis was that having a “calm” temperament would help young sexually naïve ewes cope with this novel and potentially stressful situation and so improve their response to the “ram effect.” The neuroendocrine responses of all ewes were quantitatively the same although the adult experienced ewes had a faster neuro-endocrine response compared to young sexually naïve ewes independent of their temperament. Contrary to our expectation, fewer “calm” sexually naïve ewes ovulated after the ram effect; 18% compared 62% for “nervous” ewes and 100% in adults of either temperament. Being “nervous” seems to have helped the young sexually naïve ewes respond to the “ram effect,” possibly because they were more alert and attentive toward the male. In another experiment exposure to a series of different acute stressors for the 2 days before and after the “ram effect” which increased cortisol levels decreased the proportion of young sexually naïve Ile de France ewes ovulating (Chanvallon et al., 2010b), although the short-term LH responses were not affected. The causes of these breed differences is unknown but in several species (rat, mice, pig, quail, human) the response to stress has a genetic component (Eley and Plomin, 1997; Mormede et al., 2011) and this may also apply to sheep.

## Neural circuitry involved in the ram effect

Socio-sexual cues, similar to other factors that modulate reproduction (nutrition, stress, photoperiod), act on central nervous system networks that ultimately converge on the GnRH neurons and in this way modulate the activity of the HPG axis. According to Herbison (2006) the activity of each GnRH neuron could be affected by approximately 5 million neurons, so it seems a big challenge to understand how information derived from external cues such as the “ram effect” can have such a specific effect.

Most work has focussed on establishing the neural circuitry within the hypothalamus and preoptic regions which is critical for the male effect. In sheep, as in most mammals, GnRH is released in a pulsatile fashion with each pulse of GnRH inducing a pulse of LH which is the parameter generally analyzed in neuroendocrine studies. This pulsatile secretion of GnRH is regulated by gonadal steroids at the level of the mediobasal hypothalamus (Knobil, 1981; Karsch, 1984; Maeda et al., 2010; MBH) by mechanisms that are not yet clear (Tsutsumi and Webster, 2009). Studies from a Japanese group have correlated, multiunit electrical activity of neurons in the MBH with the LH response to male goat odor in ovariectomized females (Mori et al., 1991; Hamada et al., 1996; Murata et al., 2014) and this area of the brain is also thought to be central to the male effect in sheep (De Bond et al., 2013; Ohara et al., 2014).

By contrast, the surge mode of GnRH secretion that is responsible for the preovulatory LH surge has for a long time, been considered in rodents and sheep to emanate from the preoptic area (Herbison, 2006). In rodents only a subset of GnRH neurons located in the preoptic area around the organum vasculosum lateral terminalis (OVLT) are activated during the preovulatory LH surge (Lee et al., 1992). In sheep the GnRH neurons activated during the LH surge are not preferentially localized but are scattered throughout the entire distributed field of GnRH neurons (Moenter et al., 1993). Localized implantation of estradiol has also shown that the MBH is the critical area for estradiol positive feedback in the ewe (Blache et al., 1991; Caraty et al., 1998).

The precise neural networks linking the various cues associated with the “ram effect” to the GnRH neurons are not completely established. The focus to date has mainly been on how male odor cues can influence their activity since it is clear that olfactory cues are of the great importance (Swaney and Keverne, 2009; Baum and Cherry, 2015). However, there may be some species differences particular with regard to the involvement of the main and accessory olfactory systems.

In rodents, in which most studies have been carried out, the active chemosensory cues from sexual partners are mainly detected and processed by the accessory olfactory system. Chemosensory cues from the male are detected by receptors in the vomeronasal organ and transmitted to the hypothalamus via the accessory olfactory bulb (AOB), with only one relay in the medial nucleus of the amygdala (Buck, 2000; Swann et al., 2009). Removal of the vomeronasal organs or lesioning of the accessory olfactory bulbs results in the disappearance of the effect of the partner (Beltramino and Taleisnik, 1983). However, this strict relationship between the accessory olfactory system and partner cues in rodents has been challenged by Yoon et al. (2005) who used transgenic mice to demonstrate the presence of direct projections from the main olfactory bulbs to GnRH neurons but none from the accessory olfactory system.

While much less research has been carried out in non-rodent mammals effects of olfactory cues from sexual partners appear to primarily involve air-born odors. Thus in the pig, rabbit and ferret it is the main and not the accessory olfactory system that seems to be involved in the processing of partner odor (Hudson and Distel, 1986; Dorries et al., 1997; Kelliher et al., 1999). The predominant role of the main olfactory system in processing olfactory cues from rams has been confirmed by the effects of lesions or inactivation. Destruction of the olfactory epithelium by intranasal administration of zinc sulfate or inactivation of the cortical nucleus of the amygdala by local administration of lidocaïne completely blocked the short-term LH response to ram odor (Gelez and Fabre-Nys, 2004; Gelez et al., 2004b). By contrast electro- cauterization of the vomeronasal organ, sectioning of the vomeronasal nerve or inactivation of the medial nucleus of the amygdala had no inhibitory effects, again suggesting that the accessory olfactory system is not necessary for the effect of male cues (Cohen-Tannoudji et al., 1989; Gelez et al., 2004b).

**Figure 3:**
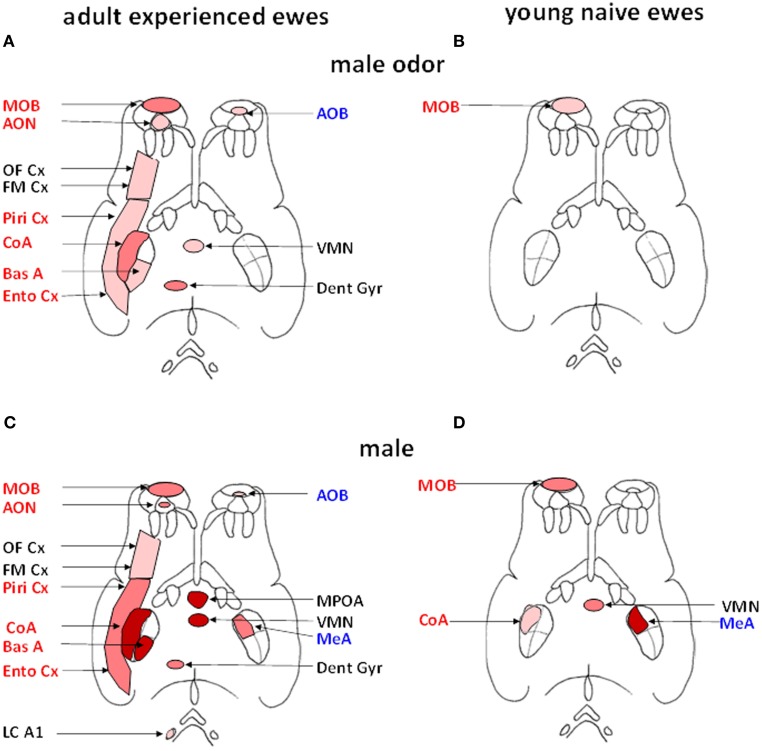
**Schematic representation of the brain regions activated (Fos immunoreactive, Fos IR) after exposure to male fleece (A, B) or to male odor (C, D) in adult ewes (A, C) or young naïve ewes (B, D)**. Regions belonging to the main olfactory system are shown in red on the left side of each panel and those belonging to the accessory olfactory system in blue on right side (adapted from Jansen et al., 1998). Within each of the four diagrams, regions shown in red are those in which the density of Fos IR cells is significantly greater than in ewes exposed to ram fleece. Regions shown in dark pink are those in which the density of Fos IR cells in treated ewes is significantly greater than in ewes exposed to the fleece of an unfamiliar ewe. Regions shown in pale pink are those in which the density of Fos IR cells is significantly higher than in ewes exposed to the test pen only. AOB, accessory olfactory bulb; AON, accessory olfactory nucleus; BasA, basal nucleus of the amygdale; CoA, cortical nucleus of the amygdala; Dent Gyr, dentate gyrus; Ento Cx, entorhinal cortex; FM Cx, frontomedial cortex; LC A1, locus coeruleus complex and A1 nucleus; MeA, medial nucleus of the amygdala; MPOA, medial preoptic area; OF Cx, orbitofrontal cortex; Piri Cx, piriform cortex; VMN, ventromedial nucleus of the hypothalamus.

Another approach that has been used to reveal the neural circuitry involved in processing male odor cues has been by through quantifying the expression of Fos protein in neurons as a molecular marker of cerebral activation (Hoffman et al., 1993). Using this approach we have shown that in adult experienced ewes male cues activate both the main and the accessory olfactory systems, although effects are much stronger within the main system (Gelez and Fabre-Nys, 2006). These findings are illustrated in Figure 3. In the main olfactory bulb, the cortical nucleus of the amygdala and the hippocampal dentate gyrus activation is relatively selective since Fos expression was increased to a greater extent after exposure to ram fleece than after exposure to female fleece (Figure 3A). In the piriform and entorhinal cortices, that are relays of the main olfactory system (Kevetter and Winans, 1981; Jansen et al., 1998), male and female odors induced higher Fos expression than the control situation, suggestive of a more general response to socio-sexual stimulation. In the accessory olfactory bulb on the other hand while Fos expression was higher after exposure to male odor than after the control situation it showed a similar response to female odor, indicating that the accessory olfactory system may respond more generally to social odors rather than in selective detection of those associated with males. These results are however challenged by a recent study which found Fos activation in the accessory but not the main olfactory system of St Croix ewes in response to extracts of hair from male goats that induce an increase in LH secretion (Ohara et al., 2014). However, the physiological relevance of these intriguing results remains to be established. Interestingly male odors also activate a number of other brain regions known to have more generalized roles in cognitive, emotional and reproductive functions including the basal amygdala, frontomedial cortex and ventromedial nucleus of the hypothalamus. Thus male odors may potentially influence a range of female behavioral responses as well as the gonadotropic axis.

Specific activation of the hippocampal dentate gyrus by male odor together with the olfactory bulb (Gelez and Fabre-Nys, 2006) is indicative of either formation and/or recall of a social recognition memory since both regions are known to play a key role in this respect (Brennan and Kendrick, 2006; Sanchez-Andrade and Kendrick, 2009). Contact with a ram has also been reported to stimulate greater cellular proliferation in ewes compared to when they are left alone (Hawken et al., 2009b). The main source of axons to the dentate gyrus is the perforant path that arises from the entorhinal cortex and intrinsic connections with the rest of the hippocampus (Treves et al., 2008). Thus differential processing of odor cues from individual rams by olfactory and hippocampal regions may in turn impact on the ultimate effects that their individual cues have, or simply whether they are familiar or not, on the subsequent activitation of GnRH neurons.

We have also found that experience modifies the extent of activation within the olfactory system in response to male odor cues. Thus, exposure to rams has very limited effects in young and sexually naïve ewes by comparison with experienced ones (Chanvallon and Fabre-Nys, 2009). In these inexperienced ewes the only region more strongly activated by male than by female odors is the first relay of olfactory inputs, the main olfactory bulb (Figure 3B). Thus in the absence of any previous experience with rams, the link between their specific olfactory cues and limbic and hypothalamic regions mediating effects on GnRH neurons and reproduction are absent or weak, although the animals are capable of discriminating their odors.

As discussed above, actual contact with rams is generally more potent than simple exposure to their fleece in inducing the “ram-effect.” In support of this it has been found that such direct contact with a ram does indeed induce greater activation in many brain regions in contrast to exposure to male odor alone, or female odor, or control situations. Regions showing such enhanced activation include the preoptic area (MPOA), the ventromedial nucleus (VMN), the medial (MeA), cortical (CoA) and basal nuclei of the amygdala (Figure 3C). Importantly, destruction of the olfactory epithelium or its inactivation by lidocaine fail to prevent an increase in LH pulsatility when adult ewes are exposed to direct contact with a ram (Gelez et al., 2004b). Thus it would appear that male cues involving other sensory modalities must be contributing in some way. However, to date we have found no evidence for activation in the visual association cortex of anestrus animals in response to visual cues from rams (Gelez and Fabre-Nys, 2006) in agreement with the limited effect of exposure to images of rams on LH secretion in anestrus ewes (Hawken et al., 2009a). This is in contrast to the situation in estrus when association visual cortex is activated (Ohkura et al., 1997) and visual cues from ram faces can induce neurochemical changes in the MBH (Fabre-Nys et al., 1997). Similarly we have found no evidence for activation in auditory brain regions.

The enhanced neural activation pattern seen in response to actual male cues as opposed to odor cues alone also shows an impact of experience. Thus in young and sexually naïve ewes Fos expression changes were only detected in the main olfactory bulb, the cortical and medial nuclei of the amygdala and the ventromedial nucleus of the hypothalamus (Figure 3D) (Chanvallon and Fabre-Nys, 2009). This is more extensive than seen in response to male fleece, where only activation in the olfactory bulb was found. Indeed, the most notable difference between responses to the ram, as opposed to only its fleece, in naïve animals was in the strength of olfactory bulb activation. An important role of experience may therefore be to enhance the response of the olfactory bulb to male cues resulting in a more extensive pattern of activation in cortical, limbic and hypothalamic regions. This experience-dependent increased activation in the olfactory bulb may reflect learning of odor (Shea et al., 2008; Sanchez-Andrade and Kendrick, 2009; Tong et al., 2014) and other characteristics of rams and strengthened interactions with other downstream projection regions.

An increasing amount of research has focussed on hypothalamic and preoptic region circuitry involved in translating the information from male cues conveyed by projections from olfactory and limbic regions. Direct contact with a ram or exposure to ram odor increases the percentage of GnRH cells expressing Fos in the POA and OVLT of adult experienced ewes (Gelez and Fabre-Nys, 2006) but not in young naïve ones (Chanvallon and Fabre-Nys, 2009). This is clearly consistent with the different responses to the “ram effect” observed in experienced and sexually naïve ewes. Although increased Fos expression also occurs in the hypothalamic ventromedial nucleus after exposure to either the ram or its odor, inactivation of this structure has no effect on the LH response to either ram odor or the ram itself. This suggests that this region, which is also activated in naïve ewes, may play a more general function in responding to cues from the ram, possibly related to increased attention (Gelez and Fabre-Nys, 2004).

An important question is therefore whether information from male odor and other cues is relayed directly to GnRH neurons, or by a more indirect route? In mice there is a direct connection between the main olfactory bulb and GnRH neurons (Yoon et al., 2005), so it is possible that part of the activation of GnRH neurons observed in ewes is due to such a direct connection, although this has yet to be established. On the other hand, in recent years many studies have shown that kisspeptin neurons have a major role in the control of GnRH secretion, and provide an important link between the GnRH neurons and sex steroids. Kisspeptin neurons may also mediate the effects of many other factors which influence the HPG axis (see review by Pinilla et al., 2012).

So are kisspeptin neurons the target for olfactory and limbic system projections involved in processing male cues important for mediating the “ram effect?” In sheep, as in other species, there are two populations of kisspeptin neurons, one in the preoptic area and another in the arcuate nucleus (Franceschini et al., 2006; Mikkelsen and Simonneaux, 2009). It was first considered that the two populations had different physiological roles (see review by Lehman et al., 2010); the preoptic area population of kisspeptin neurons in the LH surge and the arcuate population in the control of the pulsatile secretion of GnRH. However, a recent study has shown that in sheep the arcuate population of kisspeptin also has a role the LH surge (Merkley et al., 2012).

A potential direct functional role for kisspeptin neurons in mediating the “ram effect” is indicated by the finding that intracerebroventricular (ICV) administration of a kisspeptin antagonist (P271) 1 h before the introduction of rams prevents the increase in pulsatile LH secretion in response to them (De Bond et al., 2013). Furthermore, an increased proportion of kisspeptin neurons express Fos after ewes are exposed to a ram as opposed to an unfamiliar ewe (Ghenim et al., 2012), or not exposed to any other sheep (De Bond et al., 2013). Both populations of kisspeptin neurons seem to be involved and exposure to a ram for 2 h (Ghenim et al., 2012) or 3 h (De Bond et al., 2013) results in a 10 fold increase in the proportion of kisspeptin neurons expressing Fos in the arcuate nucleus. We also found an increase in the preoptic area although it was less marked (38%) than in the arcuate population, with 71% of the kisspeptin neurons expressing Fos-IR after 2 h of contact with a ram (Ghenim et al., 2012). De Bond et al. (2013) on the other hand failed to detect any preoptic changes. The recruitment of kisspeptin neurons in both locations is rapid and does not change significantly when the duration of exposure to rams is extended to 12 h. This suggests that the kisspeptin neurons become involved very soon after the first contact between the sexual partners. The involvement of kisspeptin neurons may also last for some time because Fos protein normally disappears a few hours after transient stimulation (Hoffman et al., 1993), whereas in our experiment 38% of kisspeptin neurons in the arcuate population and 67% in the preoptic area population still contained Fos-IR in ewes exposed to the ram for 12 h (Ghenim et al., 2012).

Thus while the precise neural circuitry involved in mediating the ram effect requires further confirmation, a working hypothesis at this stage is that odor cues from males are processed primarily by a core circuit involving the main olfactory bulb and cortical amygdala and influence both preoptic and hypothalamic kisspeptin neurons which in turn then activate GnRH neurons (see Kendrick, 2014). Kisspeptin and/or GnRH neurons may also be more indirectly influenced by other cortical, limbic and hypothalamic regions responding to olfactory or other cues produced by the male.

## Classical neurotransmitters involved

The classical neurotransmitter systems via which socio-sexual stimulation modulates GnRH neurons are only poorly understood. The best documented is the noradrenergic system which is involved in male-stimulated ovulation in rabbits and ferrets (Wersinger and Baum, 1997; Yang et al., 1996, 1997). In rabbits for example there is a parallel increase in noradrenalin (NA) and GnRH in the MBH within 10 min of mating (Kaynard et al., 1990). Furthermore, ICV infusions of the α1 adrenergic receptor antagonist prazosin, or administering it directly into the arcuate median eminence, either suppressed or reduced the post-coital GnRH and LH surge surges (Yang et al., 1998).

**Figure 4:**
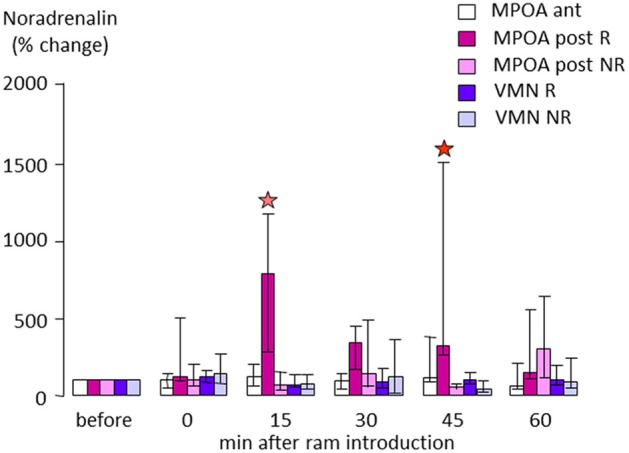
**Changes in extracellular noradrenaline concentration in the anterior (ant) or posterior part (post) of the medial preoptic area (MPOA) or in the ventromedial nucleus (VMN) of anestrus ewes that either responded (R) or did not respond by an increase in LH pulsatile secretion when exposed to a ram (NR)**. Samples were collected by microdialysis every 15 min and measured by electrochemical detection as in Fabre-Nys et al. (1994). Data are presented as percentage changes compared to the mean concentration during the three samples before male introduction. Data were compared using Friedman analysis of variance followed (when *p* < 0.05) by Dunn's multiple comparisons test a red star indicates that the post-treatment mean is different from the pretreatment mean (*p* < 0.05) and a pink star that it tended to be different (*p* = 0.054).

In sheep, when anestrus ewes are exposed to rams the rapid increase in LH is paralleled by a nearly10-fold increase in NA concentrations in the posterior part of the medial preoptic area (MPOA, Figure 4), suggesting that evoked NA release in this region may be influencing GnRH neurons to promote subsequent LH release (Fabre-Nys et al., 2005, Supplementary Material). However, interestingly when estrus ewes are exposed to a ram, or just to a picture of its face, increased NA release is observed in the MBH rather than the MPOA (Fabre-Nys et al., 1994, 1997), although the onset of increased concentrations together with their amplitude and duration are very similar in the two contexts. These findings overlap to some extent with those in the rabbit where post-coital GnRH surges in females are also associated with increased NA release in the MBH but not in the anterior hypothalamus (Kaynard et al., 1990). Thus there may be some subtle differences in the ways that male cues alter LH release when animals are in anestrus as opposed to estrus, possibly associated with differing reproductive hormone profiles.

Direct manipulation of the noradrenergic system in the posterior MPOA during the ram effect using localized retrodialysis infusions has further confirmed its role in the response to male cues (Fabre-Nys and Scaramuzzi, 2014, Supplemetary Material). Infusion of NA into the posterior preoptic area increased the proportion of ewes responding to a handful of ram fleece that by itself had a sub-threshold stimulating effect (10/11 vs. 5/10 animals). The frequency of LH pulses was also increased following NA infusions whereas the ram odor alone had no significant effects in control ewes. Infusion of the α1 antagonist Prazosin in the posterior MPOA did not affect the proportion of ewes responding to the “ram effect” (7/11 vs. 8/11), and LH pulse frequency was increased both in control and Prazosin-treated ewes. However, the frequency and the amplitude of LH pulses following the “ram effect” were significantly reduced following Prazosin compared to the controls. These findings are somewhat similar to those in the rabbit where Prazosin infusions into the arcuate median eminence region only attenuated post-coital GnRH and LH surges, whereas ICV infusions completely suppressed them (Yang et al., 1998). Thus it is possible that NA is acting at multiple sites via the α1 receptor to influence LH secretion and ovulation both in the context of male-induced ovulation in female rabbits, and the ram effect in anestrus ewes.

In sheep, as in other species, noradrenergic neurons have their cell bodies within the pons and medulla of the brainstem (Tillet and Thibault, 1989) and some of them project to the MPOA and the MBH (Tillet et al., 1993). The proportion of tyrosine hydroxylase (TH), the rate limiting enzyme for the synthesis of NA, immunoreactive cells that were also immunoreactive for Fos in the A1 and in the locus coeruleus (LC) complex (A6–A7) is higher in ewes exposed to a ram than in controls (Fabre-Nys and Scaramuzzi, 2014, Supplementary Material). This suggests that noradrenergic afferents are involved in the response to male cues in anestrus ewes, in a similar way to that observed in rabbits and ferrets after mating (Kaynard et al., 1990; Wersinger and Baum, 1997; Yang et al., 1997, 1998). The noradrenergic projection from the LC to the olfactory bulb has been shown to be important in olfactory recognition memory (Brennan and Kendrick, 2006; Sanchez-Andrade and Kendrick, 2009) and may also play a role in increasing sensitivity of the olfactory bulb to weak odors (Jiang et al., 1996). Furthermore, in the context of maternal ewes recognizing their lambs there is an experience dependent enhancement of olfactory bulb NA release (Lévy et al., 1993). Locus coeruleus stimulation has also been shown to augment MPOA stimulated GnRH release in rodents (Gitler and Barraclough, 1987). Thus in the context of odor stimuli from rams the LC noradrenergic projections to the olfactory bulb and to the MPOA may play a key role both in experience dependent perception and recognition of these odors at the level of the olfactory bulb and in facilitating GnRH release at the level of the MPOA. Importantly, since the LC is also associated more generally with relaying arousal and autonomic changes to widespread regions of the forebrain (Sara and Bouret, 2012), it is in a position to signal more general responses to the actual presence of a ram beyond those relating to specific odor molecules from its wool alone. Thus the LC projections may play a role in mediating more general influences of male cues on both reproductive and odor processing functions.

An important unanswered question at this stage is clearly how altered noradrenergic signaling modulates the activity of GnRH neurons (Herbison, 1997; Goodman et al., 2002; Clarke et al., 2006; Szawka et al., 2013). If inputs to the hypothalamic and preoptic area conveying information about ram cues involve noradrenergic signaling then one might expect from our above discussion of the potential neural circuitry mediating the ram effect that there would be some interaction with kisspeptin neurons. While this has not been shown directly a recent report on *Kiss1* knockout rats has shown that they fail to show LH release in response to either noradrenergic or glutamatergic stimulation (Uenoyama et al., 2015). Thus noradrenergic involvement in the ram effect might indeed be partly via an interaction with kisspeptin signaling, although further studies are clearly required to establish this.

## Potential scenarios of events during the ram effect

**Figure 5:**
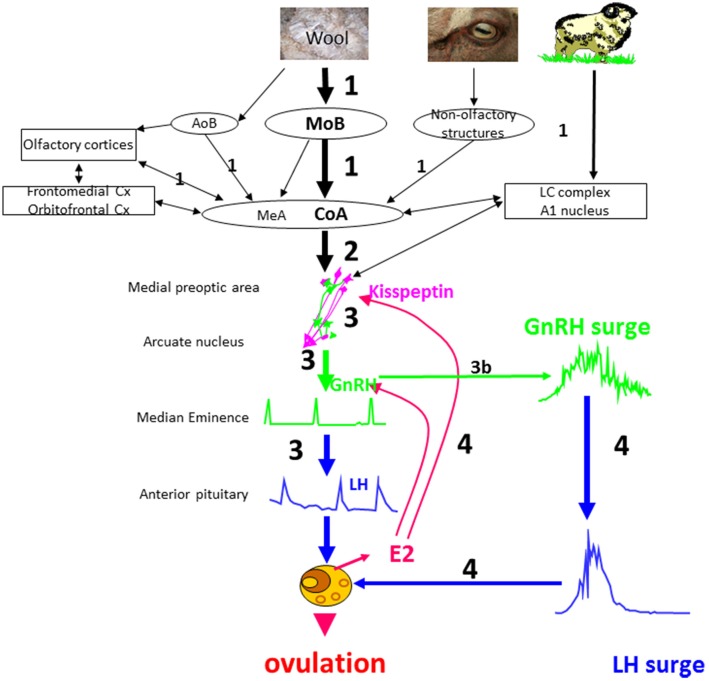
**Schematic illustration of a proposed model for the sequence of neuroendocrine events in ewes following the “ram effect”**. The numbers indicate the chronology of events and their size the relative importance of a specific step. In a small proportion of ewes the step 3 leads directly to a LH surge (step 3b) without the need for estradiol positive feedback. MOB, main olfactory bulb; AOB, accessory olfactory bulb; CoA, corticomedial nucleus of the amygdala; Cx, cortex; MeA, medial nucleus of the amygdala; LC, locus coeruleus complex.

The results obtained so far allow us to suggest a model of the sequence of events when anestrus ewes are exposed to a sexually active rams (Figure 5).

*Step 1:* The ram using principally odor cues and socio-sexual behavior patterns activates the main olfactory bulb and to a lesser extent the accessory olfactory system, of the courted ewe. This activation is transmitted along relays in the amygdala and the associated cortices (piriform, entorhinal cortices) and also to several cortical areas that have broader “cognitive “functions in terms of associative learning (orbitofrontal and frontomedian cortices) and to the noradrenergic system. Activation of these structures depends on the ewe's previous experience with rams and the olfactory bulb and dentate gyrus probably have an important role in learning or remembering both male cues in general and also those of specific individuals.
*Step 2:* The brain regions that have been activated by ram cues in turn stimulate the GnRH network at least partly via activation of kisspeptin neurons. Factors such as stress or nutrition might also interact at this level to modulate the activation of the GnRH network.
*Step 3:* Activated GnRH neurons secrete GnRH that induces short-term pulsatile secretion of LH. Most ewes will show this response but the frequency and amplitudes of the pulses can be modulated by several environmental factors; stress, nutrition, photoperiod and socio-sexual experience, and also by variation in the sensitivity of the gonadotrophic axis to estradiol feedback. The increased pulsatile secretion of LH stimulates ovarian follicles to secrete estradiol. In some ewes there are no follicles mature enough to respond to LH and so they will not secrete sufficient estradiol to induce a LH surge. In these ewes the action of the “ram effect” will be arrested at this stage.
*Step 3b:* In some ewes exposure to a ram immediately induces a LH surge (“precocious” LH surges) without the need for a period of increased pulsatile secretion of LH. We suggest that NA is involved in the induction of “precocious” LH surges. Ewes with higher activity of the noradrenergic system or a higher sensitivity to noradrenergic inputs may be more likely to have “precocious” LH surges.
*Step 4:* Estradiol secreted by responsive follicles stimulates preovulatory secretion of GnRH and LH surges as occurs in cyclic animals, leading to a LH surge and ovulation. Differences in sensitivity to estradiol result in ewes displaying different latencies in terms of the onset of their LH surge.

## What the ram effect could tell us about the HPG axis

In many species, social interactions are major contributors to the adaptation of reproduction to a changing environment. This is the case for sheep and many other ungulate species in which the introduction of a male into a group of females in a reproductively quiescent state will reinstate cyclicity. These socio-sexual effects act by the modulation of GnRH secretion. In this last section we will propose a few questions that could be addressed either directly or indirectly using the “ram effect” as the experimental model in a new and fruitful way.

### Pulsatile secretion of GnRH

A Japanese group has already shown in very elegant studies in goats that the effects of male stimuli in goats can help unravel the details of pulsatile GnRH secretion (Hamada et al., 1996; Ichimaru et al., 2008). Their techniques have been developed using a miniature Shiba goat. However, the sheep is a species in which it is possible to study GnRH secretion directly by sampling hypothalamo-pituitary portal blood (Caraty and Locatelli, 1988). Because of the over-riding significance of ovarian steroids in the control of pulsatile GnRH secretion most studies have focused on this. The “ram effect” modulates pulsatile LH secretion and so probably also the pulsatile secretion of GnRH, although this has never been tested.

The “ram effect” is most potent when ewes are anestrus and more sensitive to the influence of negative estradiol feedback (Goodman et al., 1981, 2002). Furthermore, rams can also influence reproductive neuro-endocrine function in ewes during the luteal phase when some progesterone is present, although this steroid is known to have a very strong negative feedback effect on GnRH pulsatile secretion (Goodman, 1996; Goodman et al., 2011). So clearly exposure to male cues can in some situations override the negative feedback effect of steroids. The mechanisms involved are currently unknown but it would be interesting to test if the control of GnRH secretion by ram cues is through a different neural network to that involved in negative feedback. Experiments in mice have identified a direct link between male odor and GnRH neurons (Boehm et al., 2005; Yoon et al., 2005), although demonstrating such a direct link in ewes may be difficult. Furthermore, considering the number of brain regions that are activated when ewes are exposed to rams it is very likely that many more of them could have direct or indirect links with the medial preoptic area and the GnRH neuronal network than are known currently. Studies on the “ram effect” could help discover these unknown connections.

### The LH surge

The induction of a LH surge by exposure of anestrus ewes to a ram is nearly always due to an increase in estradiol secretion. The mechanism involved is most likely the same as the one that induces the preovulatory LH surge in spontaneous ovulators. However, in a few ewes the LH surge is induced immediately after rams are introduced suggesting a different mechanism and one closer to that in induced ovulators (Fabre-Nys et al., 2013). Several authors have suggested that the dualistic theory of spontaneous or induced ovulation is an over simplification and that both systems could coexist in most females. To date very few attempts have been made to test this hypothesis and to unravel the two types of neural circuitry that would be required to explain the duality of induced and spontaneous ovulation. The identification of factors associated with “precocious” LH surges could help identify these neural circuits.

### Role of noradrenalin

Noradrenalin is clearly involved in the male induced LH surge in induced ovulators but has a more “permissive” role in the control of GnRH secretion in spontaneous ovulators. Direct connections between GnRH neurons and NA terminals have been described in many species and since GnRH neurons possess noradrenergic receptors the action of NA on them could be direct. However NA is released in a variety of circumstances and mediates changes both in attention and general arousal. The mechanisms of these effects are not clear and the hypothesis that some of these actions could take place though the kisspeptin neurons has not been tested, although this would be an interesting possibility.

### Study of the impact of environmental factors

A number of environmental factors (e.g., nutrition or stress) can modulate the response to the “ram effect” and in many cases the target of this modulation is the pulsatile secretion of GnRH. Indeed, some factors are known to have direct modulatory effects on the GnRH network (Chand and Lovejoy, 2011; Roa and Tena-Sempere, 2014) and the mechanisms involved are starting to be understood, although, exactly how these factors interact with the effect of the male is not known. Understanding these interactions would help provide a more thorough understanding of the impact of environmental factors on reproduction.

### Effect of experience

Young ewes that are sexually naive have weaker physiological responses to the ram or to ram odor and the socio-sexual cues from the ram are less able to activate brain regions than in sexually experienced ewes. The physiological response of sexually naïve ewes to the ram is enhanced if the ewes have had previous contact with sexually mature rams so that they could “learn” their characteristics. In a physiological sense, exactly what is meant by “learned” and how can sexual experience modulate the GnRH network? To our knowledge, this has never been studied, but the “ram effect” would certainly be a useful and potentially fruitful model to address this question.

### Conflict of interest statement

The authors declare that the research was conducted in the absence of any commercial or financial relationships that could be construed as a potential conflict of interest.
