# Supplementary material for: The “ram effect”: new insights into neural modulation of the gonadotropic axis by male odors and socio-sexual interactions
Source: Front Neurosci. 2015 Apr 9;9:111. doi: 10.3389/fnins.2015.00111 (PMC4391029; doi:10.3389/fnins.2015.00111)
Supplement: Supplementary file 1 [file DataSheet1.PDF]

Noradrenaline concentrations in the hypothalamus of anoestrus ewes following the ram-induced luteinizing hormone release

Claude Fabre-Nys[a] and Keith M. Kendrick[b]

Sheep are seasonal breeders, but exposure of anoestrus ewes to rams results in a rapid increase in luteinizing hormone (LH) secretion, eventually leading to surge in LH. Although LH secretion is known to be under the control of many neurotransmitters, noradrenaline (NA) is of particular importance for the LH surge in induced ovulators, although little is known about its role in LH secretion induced by males in spontaneous ovulators. To address this question, anoestrus ewes fitted with guide-tubes in the medial preoptic area (MPOA) or the ventromedial hypothalamus were subjected to microdialysis and blood sampling every 15 min for an hour before and 2 h after exposure to rams, and the concentrations of LH, monoamine and amino acid AQ4 **transmitters were measured. In ewes implanted in the posterior MPOA that responded to the ram by an increase in LH pulses, NA concentrations changed after exposure to the ram ($P < 0.018$) and were higher at 15 ($P < 0.054$) and 45 min ($P < 0.03$) after male introduction than before. By contrast, no change in NA could be detected in ewes implanted in the same region, but not responding to the ram, or in those showing increased LH pulsatility, but implanted in the anterior MPOA or in the ventromedial hypothalamus. No changes were observed in other neurotransmitters or when the ewes were exposed to male odour alone. These results suggest that NA release in the posterior MPOA is selectively involved in the triggering of LH secretion by rams in anoestrus ewes. *NeuroReport* 00:000–000 Copyright © 2015 Wolters Kluwer Health, Inc. All rights reserved.**

NeuroReport 2015, **00**:000–000

Keywords: luteinizing hormone, medial preoptic area, microdialysis, neurotransmitter, noradrenalin, ram effect, sheep, ventromedial hypothalamus

[a]UMR 7247 Physiologie de la Reproduction et des Comportements, CNRS, INRA, Université de Tours, Institut français du cheval et de l'équitation, Nouzilly, France and [b]Key Laboratory for Neuroinformation, Center for Information in Medicine, University of Electronic Science and Technology of China, Chengdu, China AQ2

Correspondence to Claude Fabre-Nys, PRC Centre INRA Val de Loire 37380 Nouzilly, France AQ3
Tel: +33 2 47 42 79 75; fax: +33 2 47 42 77 43;
e-mail: claude.fabre@tours.inra.fr

Received 26 February 2015 **accepted** 5 March 2015

Introduction

AQ5 Reproduction in mammals is controlled by the interplay between the hypothalamus secreting gonadotropin releasing hormone (GnRH), the pituitary secreting luteinizing hormone (LH) and follicular stimulating hormone (FSH) and the production of sex hormones by the gonads. In females, these hormones control the growth, differentiation and ovulation of follicles from the ovary that produce steroids, which in turn regulate GnRH, LH and FSH secretion through positive and negative feedback. Reproduction is also under the influence of a variety of environmental factors such as sociosexual interactions. Depending on the context, these interactions can result in total inhibition or delay of reproduction or, on the contrary, in its stimulation and synchronization [1]. Most extreme examples are, on the one hand, eusocial mole rats in which only one female, the 'queen' and a few males can reproduce [2] and, on the other, rabbits and ferrets, where ovulation does not occur in isolated animals [3].

Sheep are seasonal breeders, but exposure of ewes in seasonal anoestrus to sexually active rams results in a rapid increase in LH secretion [4]. The stimulation of LH secretion will eventually lead to ovulation outside of their breeding season [5]. As in many other mammalian species, olfaction plays a predominant role and most of the effect of the ram can be mimicked by the odour of its fleece [5].

Central mechanisms involved in the regulation of the hypothalamo–pituitary–gonadal axis have been studied extensively in the past decades and many different classical neurotransmitters and neuropeptides have been shown to play a role [6,7]. However, the neural pathways and transmitter systems through which sociosexual interactions modulate GnRH and LH secretion are only poorly understood. The best documented is the noradrenergic system, which is involved in the mating-induced ovulation in rabbit and ferrets [3]. In the female rabbit, the postcoital GnRH surge has been associated with an increase in noradrenaline (NA) in the mediobasal hypothalamus, but not the anterior hypothalamus [3]. Furthermore, administration of an α-adrenergic antagonist prazosin into the third ventricule of the brain prevents the induction of an LH surge by coitus [3].

In adult sexually experienced ewes, the stimulation of LH secretion by exposure to a ram during anoestrus is accompanied by an activation of the main and, to a lesser extent, the accessory, olfactory system, together with

0959-4965 Copyright © 2015 Wolters Kluwer Health, Inc. All rights reserved.

DOI: 10.1097/WNR.0000000000000367

several limbic and cortical structures and of hypothalamic GnRH and kisspeptin neurons [8–10]. However, it has not been established which neurotransmitters may be involved in the activation of GnRH neurons in this context.

In this study, we have therefore used an in-vivo microdialysis sampling approach to measure changes in classical neurotransmitter known to be involved in the control of LH secretion [6,7] and occurring during interaction with rams during anoestrus in both the medial preoptic area (MPOA), where most GNRH cells are located, and in the ventromedial hypothalamus (VMH), where we had shown previously that NA concentrations are increased when the oestrus ewes are exposed to males [11]. To establish specific associations between observed neurotransmitter changes and the effect of rams on LH, we compared release profiles in animals that did, or did not, show increased LH pulse frequency.

Materials and methods

Animals

All the experiments were conducted on adult Ile-de-France ewes (2–7 years old) under a natural photoperiod during anoestrus (March–July) at the INRA station in Nouzilly. Females were kept indoors and had no contact with rams for at least 2 months before the beginning of the experiments. They were fed daily with a constant diet of straw, lucerne pellets and mineral supplement and had free access to water. Sexually experienced adult Ile-de-France rams were used to provide the male stimulus. For odour stimuli, wool was collected from the fleece of several males from a range of different breeds and stored in sealed bags at − 20°C.

The ewes were confirmed as seasonally anovulatory by establishing that they showed a pattern of persistently low plasma concentrations of progesterone (< 1 ng/ml) in blood samples collected weekly [12]. This indicated the absence of functional corpora lutea.

All experimental procedures were carried out in accordance with French and European regulations on care and welfare of animals in research, with authorization of the French Ministry of Agriculture and with local ethical approval (authorization No. 006259).

Brain surgery

Ewes were fitted bilaterally with guide cannula (Procedure needle 18G; Becton Dickinson, Heidelberg, Germany) aimed at 5 mm above the target anterior (MPOA ant $n = 9$) or posterior MPOA (MPOA post $n = 19$) or VMH ($n = 17$) using a technique that combined a stereotaxic method and lateral and frontal radiography established by Fabre-Nys *et al.* [13]. The stereotaxic coordinates were determined using an atlas [14] and our own data [13]. Surgery was carried out under general anaesthesia induced by an injection of thiopental (1 g nesdonal; Specia Rhone Poulenc, Paris, France) and atropine sulphate (20 mg; Lavoisier, Paris, France) and maintained by closed-circuit halothane (Bélamont, Neuilly, France). Full aseptic precautions were taken throughout. After surgery, ewes were injected with 5 ml of dexamethasone (Diurizone; Vetoquinol, Lure, France) daily for 3 days. Females were allowed a minimum of 2 weeks of recovery before sampling and during this period, they were habituated to handling.

Microdialysis sampling and assays

Microdialysis probes (Mab 6, 5 mm membrane length; Microbiotech, Stockholm, Sweden) were lowered into the previously implanted guide-tubes 2 h before the beginning of sampling. Krebs Ringer (pH 6.5) was pumped through the probes at 2 μl/min using a battery-driven syringe pump (MS16A; Graseby Medical, Watford, UK) taped to the animal's back. Microdialysates and blood samples were collected every 15 min for an hour before and 2 h after exposure to rams (all groups) or its fleece (MPOA post and VMH groups) into 500 μl eppendorf tubes containing 5 μl of 2% hydrochloric acid. Microdialysis samples were frozen (− 20°C) before analysis of monoamine (noradrenaline, dopamine and serotonin) concentrations by microbore HPLC with electrochemical detection as described previously [15], detection limits = 100 − 200 pM). Aspartate, glutamate, citrulline, arginine, taurine and GABA were measured by HPLC with precolumn derivatization with *o*-phthaldialdehyde and fluorescence detection (detection limit = 1 –5 nM) [15]. For a given animal and a given session, concentrations were only measured on one side of the brain.

Blood samples and luteinizing hormone assays

Blood samples were collected every 15 min for 1 h before and 2 h after exposure to the ram or its fleece through catheters inserted into their jugular vein the day before the experiment. Samples were centrifuged and plasma were stored at − 20°C until assayed. Plasma concentrations of LH were determined using an enzyme-linked immunoassay [16]. The sensitivity of the assay was 0.1 ng/ml and the intra-assay and interassay coefficients of variation for reference samples were 9.3 and 5.2%, respectively.

Postmortem histology

At the end of the experiment, all the females were euthanized by an anaesthetic overdose and their brains were removed for subsequent histology. Free-floating frontal sections (40 μm thick) were cut on a freezing microtome and stained with cresyl violet to facilitate histological identification of probe location.

Data analysis

LH pulses were identified as a rapid increase in concentration exceeding three times the standard deviation

of the baseline reference (0.4 ng/ml), followed by an exponential-type decrease. An animal was classified as responding if the frequency of LH pulses after the exposure to the ram or its fleece was greater than that occurring before.

Some animals had to be excluded from analysis because of problems during the sampling or where more than two samples were missing. Microdialysis data were analysed separately for each implantation site (MPOA ant, MPOA post and VMH) and condition (male, odour) in animals showing an LH response to the male (MPOA ant R, $n=7$; MPOA post R male, $n=7$ and MPOA post R odour $n=8$, VMH R male $n=7$, R odour $n=10$) or those that did not show a response (MPOA post male NR, $n=7$, MPOA post odour NR, $n=6$). Because of the low number of animals failing to respond with MPOA ant ($n=1$), VMH male ($n=5$) and VMH odour ($n=4$) placements, the MPOA ant NR data were excluded from analysis and VMH nonresponders were combined ($n=9$).

To control for individual differences in the basal concentrations of neurotransmitters, the effects of exposure to the male or its fleece were expressed and analysed statistically as percentage changes relative to the mean concentration of the baseline samples collected before stimulation (three samples). T0 corresponds to the samples collected while the male waited to be introduced into the female's pen. If one sample was missing, a value of 100 (baseline) was assigned to it to allow statistical analysis to be carried out. Because the data were not normally distributed, the statistical analysis of transmitter changes after exposure to the ram or its fleece was carried out according to implantation site and type of response using a Friedman's analysis of variance for related samples, followed, when significant ($P<0.05$), by paired comparisons with the baseline level before the male stimulus by Wilcoxon tests using a Dunn correction.

Results

The anatomical localization of the probes in the different groups is shown in Fig. 1; the median anteroposterior coordinates from the Richard atlas were MPOA ant = 33, AQ6 MPOA post = 32 and VM~~N~~ = 28, and did not differ between ewes that either responded or did not to the male cues.

NA extracellular concentrations changed significantly during the 2 h after exposure to a ram in the MPOA post of ewes responding to the ram by an increase in LH pulse AQ8 frequency (Friedman's test: 19.88, ~~dl~~ = 9; $P=0.018$; MPOA post R, Fig. 2a). The percentage changes in the NA concentration were marginally significant at 15 min ($P=0.054$), a trend was detected at 30 min ($P=0.083$) and changes were significant at 45 min ($P=0.033$) after introduction of the male. By contrast, no change in NA concentrations occurred in animals implanted in the same area but that did not show an LH response to the ram (MPOA post NR), or in responding animals implanted in the MPOA ant or in either responsive or nonresponsive animals implanted in the VMH (VMH R and VMH NR; Fig. 2a).

Although an overall analysis showed that DA extracellular concentrations changed significantly changed during the 2 h after male introduction in the group MPOA post R (Friedman's test: 17.04, ~~dl~~ = 9; $P=0.048$; Fig. 2b), post-hoc analyses did not show significant changes ($P=0.092$ and 0.084, respectively, at the 15 and 45 min time-points after male introduction). No overall significant changes were found for serotonin and for any of the amino acid neurotransmitters: aspartate, glutamate, citrulline, arginine, taurine and GABA measured after ram introduction.

Although there was some evidence for a small increase in NA concentrations in animals implanted in MPOA post and responding with increased LH pulses following exposure to male fleece alone, this failed to achieve significance (Friedman's test: 14.09, ~~dl~~ = 9; $P=0.11$; Fig. 2c). No other changes in transmitter concentrations were found after exposure to ram fleece.

Discussion

Social interactions in many species are major contributors towards the adaptation of reproduction to a changing environment. In sheep and many other ungulates, introduction of a male in a group of females in a reproductively quiescent state will induce and synchronize reproduction, which can reduce the risk of predation. Sociosexual interactions act by modulation of LH secretion [4,5]. In this study, we have shown that exposure to a ram is followed by an increase in NA extracellular concentrations in the MPOA post at 15 and 45 min time-points, but only in ewes that respond by showing an increase in LH secretion. This effect was specific to the MPOA post and did not occur in either the MPOA ant or the VMH. No significant changes were observed in concentrations of dopamine, serotonin, aspartate, glutamate, citrulline, arginine, taurine and GABA. This suggests first that interactions with the male exert very specific effects on NA neurons as observed in induced ovulators [3] and second that these neurons are involved in the LH response. It would be interesting to identify which NA neurons are involved. In induced ovulators, both the brainstem and locus coeruleus NA neurons are activated by mating [3], whereas in sheep, projections from A1 and A2 are considered the most important for the control of GnRH neurons [17,18]. However, the recent results on the role of the locus coeruleus on LH secretion in rat [19] suggest that this question should be re-examined in sheep. Exposure to male odour alone tended to have the same effect, although this did not quite achieve significance. These results suggest that NA release in the MPOA post may be involved in the LH response of ewes to ram cues during anoestrus.

Fig. 1

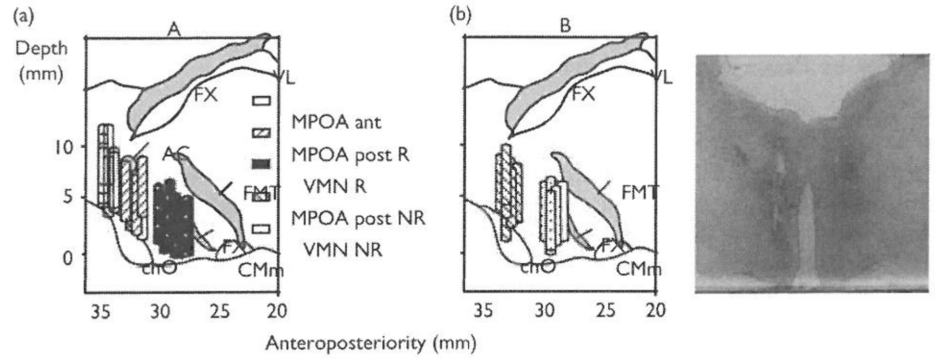

AQ7

Schematic diagram of a sagittal section through a sheep brain showing the placement of the active membrane of dialysis probes in the ewes responding (a) or not responding (b) to the introduction of a ram by an increase in LH pulses. The diameter and length of the membrane are represented on scale. AC, anterior commissure; chO, optic chiasm; CMm, mammilary bodies; Fx, Fornix; LH, luteinizing hormone.

Fig. 2

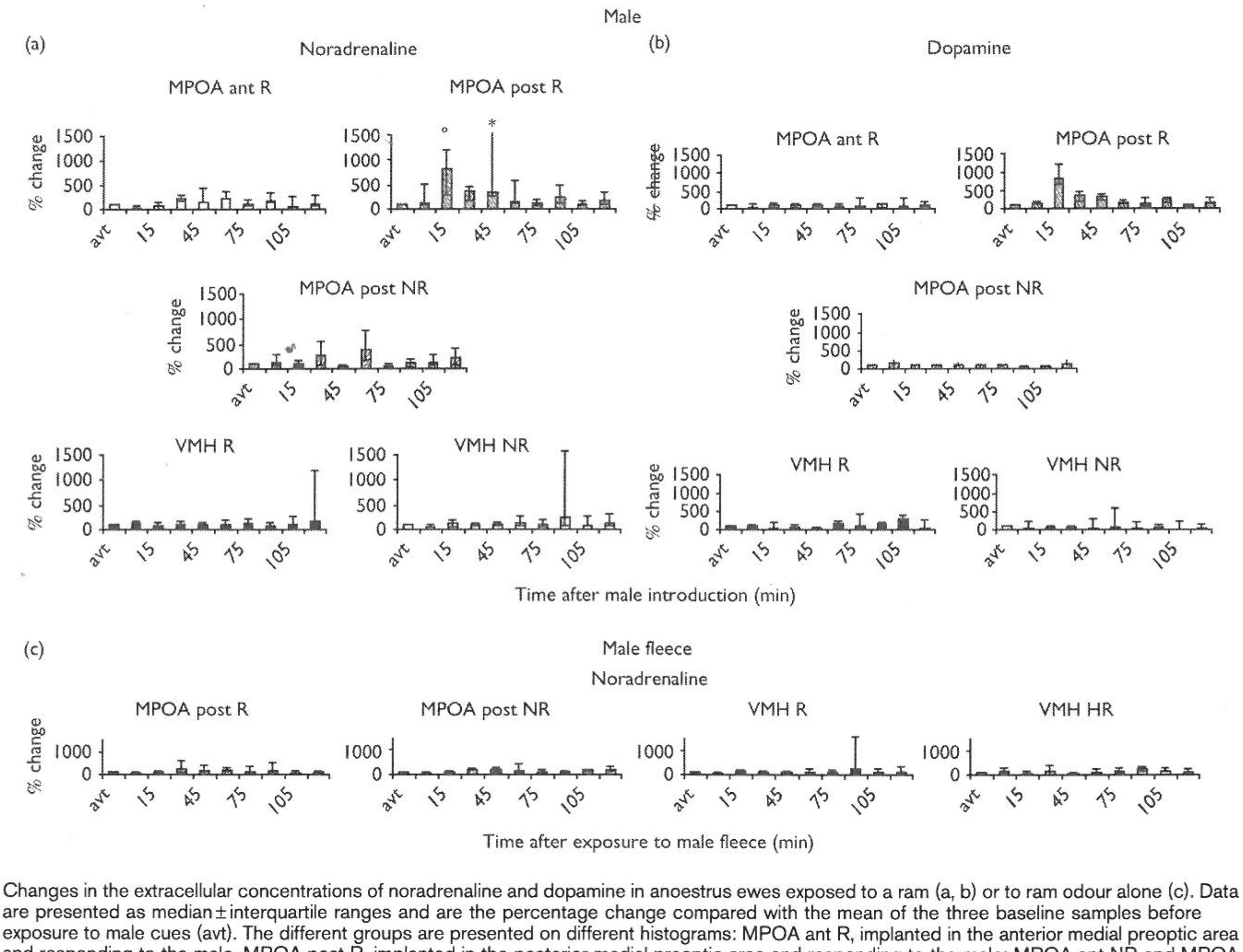

Changes in the extracellular concentrations of noradrenaline and dopamine in anoestrus ewes exposed to a ram (a, b) or to ram odour alone (c). Data are presented as median ± interquartile ranges and are the percentage change compared with the mean of the three baseline samples before exposure to male cues (avt). The different groups are presented on different histograms: MPOA ant R, implanted in the anterior medial preoptic area and responding to the male, MPOA post R, implanted in the posterior medial preoptic area and responding to the male; MPOA ant NR and MPOA post NR, same placements but not responding to the male; VMH R, implanted in the ventromedial hypothalamus and responding to the male; VMH NR, implanted in the ventromedial hypothalamus and not responding to the male. $^{\circ}P=0.054$, $^{*}P<0.05$ compared with baseline in the same group, Wilcoxon test, after a Friedman's analysis of variance established significance across each treatment group.

In induced ovulators, NA is clearly involved in the onset of the male-induced LH surge [3]. In spontaneous ovulators, the situation is less clear and the variability in NA effects on LH secretion has led to suggestions that NA may only exert a permissive effect in modulating the effect of other neurotransmitters and neuropeptides [18, 20].

During anoestrus, plasma oestradiol concentrations in ewes are very low and are considered to exert a strong negative feedback on LH secretion [21]. Exposure to a male or its odour can override this inhibitory effect of oestradiol on LH in the ewe during anoestrus. Our experiment suggests that this effect may be at least partly mediated by an increase in NA extracellular concentrations in the MPOA post influencing the activity of GnRH neurons. Close contact between NA afferent terminals and GnRH neurons has been observed in sheep [17] and α1 adrenergic receptors have been observed on GnRH neurons in rats [22]; thus, a direct action of NA on GnRH neurons is possible. However, the fact that in our study we only observed NA changes in the MPOA post, rather than in the MPOA ant, where most GnRH cells are located, suggests that NA might mainly be exerting a more indirect effect on the GnRH neuron activity. One possibility is that it could be acting by kisspeptin neurons that are scattered throughout the MPOA [23], are activated in anoestrus ewes exposed to a ram [9,10] and potently stimulate GnRH secretion [24].

In contrast to our previous observations showing that NA concentrations are increased in the VMH regions of oestrus ewes exposed either to a ram or to the picture of its face [11,25], we did not find this effect of ram cues in the current study on an anoestrus animal. The latency, amplitude and duration of LH changes were, however, very similar in the two studies. This suggests that the populations of NA neurons activated by ram cues are different during anoestrus as opposed to during the breeding season. These differences could be related to differences in the circulating level of ovarian steroids that have an effect on NA neurons and/or could be because of alteration in the dominant cue for sociosexual interactions, olfaction being the primary sense in anoestrus and vision being the most important sense during the breeding season. This requires further investigation.

Conclusion

Our study provides the first evidence suggesting that NA release in the MPOA post may play an important role in mediating increases in GnRH and subsequent LH release in anoestrus ewes exposed to rams. The 'ram effect' model may therefore provide a useful paradigm for further studies investigating NA modulation of LH release.

Acknowledgements

The authors wish to thank the staff from the INRA experimental station and hospital in Nouzilly for the care of the animals, E. Archer and H. Gelez for their help with collecting the samples and C. De la Riva for his help with neurotransmitter measurement.

Conflicts of interest

There are no conflicts of interest.

References

1 Signoret JP. Effet de la présence du male sur les mécanismes de reproduction chez la femelle des mammifères. *Reprod Nutr Develop* 1980; **20**:457–468. AQ9
2 Goldman SL, Forger NG, Goldman BD. Influence of gonadal sex hormones on behavioral components of the reproductive hierarchy in naked mole-rats. *Horm Behav* 2006; **50**:77–84. AQ10
3 Bakker J, Baum MJ. Neuroendocrine regulation of GnRH release in induced ovulators. *Front Neuroendocrinol* 2000; **21**:220–262.
4 Martin GB, Oldham CM, Cognie Y, Pearce DL. The physiological responses of anovulatory ewes to the introduction of rams – a review. *Livest Prod Sci* 1986; **15**:219–247.
5 Ungerfeld R. Socio-sexual signalling and gonadal function: opportunities for reproductive management in domestic ruminants. *Soc Reprod Fertil Suppl* 2007; **64**:207–221.
6 Barraclough CA, Wise PM, Selmanoff MK. Role of hypothalamic catecholamines in the regulation of gonadotropin secretion. *Rec Prog Horm Res* 1984; **49**:487–529.
7 Brann DW, Mahesh V. Exitatory amino acids: function and significance in reproduction and neuroendocrine regulation. *Front Neuroendocrinol* 1994; **15**:3–49.
8 Gelez H, Fabre-Nys C. Neural pathways involved in the endocrine response of anestrous ewes to the male or its odor. *Neuroscience* 2006; **140**:791–800.
9 Ghenim M, Dufourny L, Fabre-Nys C. Kisspeptin neurons are activated during the male effect in sheep. In: 2nd World Conference Kisspeptin Signaling in the Brain; 6th–9th November 2012; Tokyo, Japan. AQ11
10 De Bond JA, Li Q, Millar RP, Clarke IJ, Smith JT. Kisspeptin signaling is required for the luteinizing hormone response in anestrous ewes following the introduction of males. *PLoS One* 2013; **8**:e57972.
11 Fabre-Nys C, Ohkura S, Kendrick KM. Male faces and odours evoke differential patterns of neurochemical release in the mediobasal hypothalamus of the ewe during oestrus: an insight into sexual motivation? *Eur J Neurosci* 1997; **9**:1666–1677.
12 Canépa S, Laine AL, Bluteau A, Fagu C, Flon C, Monniaux D. Validation d'une méthode immunoenzymatique pour le dosage de la progestérone dans le plasma des ovins et des bovins. *Cahier des Techniques de l'Inra* 2008; **64**:19–30. AQ12
13 Fabre-Nys C, Blache D, Lavenet C. A method for accurate implantation in the sheep brain. In: Greenstein B, editor. *Neuroendocrine research methods, implantation and transfection procedures.* Chur, Switzerland: Harwood; 1991. pp. 295–314.
14 Richard P. *Atlas Stéréotaxique du Cerveau de Brebis* Paris, France: INRA; 1967.
15 Kendrick KM, Guevara-Guzman R, de la Riva C, Christensen J, Ostergaard K, Emson PC. NMDA and kainate-evoked release of nitric oxide and classical transmitters in the rat striatum: *in vivo* evidence that nitric oxide may play a neuroprotective role. *Eur J Neurosci* 1996; **8**:2619–2634.
16 Faure MO, Nicol L, Fabre S, Fontaine J, Mohoric N, McNeilly A, Taragnat C. BMP-4 inhibits follicle-stimulating hormone secretion in ewe pituitary. *J Endocrinol* 2005; **186**:109–121.
17 Tillet Y, Batailler M, Thibault J. Neuronal projections to the medial preoptic area of the sheep, with special reference to monoaminergic afferents: immunohistochemical and retrograde tract tracing studies. *J Comp Neurol* 1993; **330**:195–220.
18 Clarke IJ, Scott CJ, Pereira A, Pompolo S. The role of noradrenaline in the generation of the preovulatory LH surge in the ewe. *Domest Anim Endocrinol* 2006; **30**:260–275.
19 Szawka RE, Poletini MO, Leite CM, Bernuci MP, Kalil B, Mendonça LB, *et al.* Release of norepinephrine in the preoptic area activates anteroventral periventricular nucleus neurons and stimulates the surge of luteinizing hormone. *Endocrinology* 2013; **154**:363–374.

20 Herbison AE. Noradrenergic regulation of cyclic GnRH secretion. *Rev Reprod* 1997; **2**:1–6.
21 Goodman RL, Legan SJ, Ryan KD, Foster DL, Karsch FJ. Importance of variations in behavioural and feedback actions of oestradiol to the control of seasonal breeding in the ewe. *J Endocrinol* 1981; **89**:229–240.
22 Hosny S, Jennes L. Identification of alpha1B adrenergic receptor protein in gonadotropin releasing hormone neurones of the female rat. *J Neuroendocrinol* 1998; **10**:687–692.
23 Franceschini I, Lomet D, Cateau M, Delsol G, Tillet Y, Caraty A. Kisspeptin immunoreactive cells of the ovine preoptic area and arcuate nucleus co-express estrogen receptor alpha. *Neurosci Lett* 2006; **401**:225–230.
24 Messager S, Chatzidaki EE, Ma D, Hendrick AG, Zahn D, Dixon J, *et al.* Kisspeptin directly stimulates gonadotropin-releasing hormone release via G protein-coupled receptor 54. *Proc Natl Acad Sci USA* 2005; **102**:1761–1766.
25 Fabre-Nys C, Blache D, Hinton MR, Goode JA, Kendrick KM. Microdialysis measurement of neurochemical changes in the mediobasal hypothalamus of ovariectomized ewes during oestrus. *Brain Res* 1994; **649**:282–296.
