# Supplementary material for: The “ram effect”: new insights into neural modulation of the gonadotropic axis by male odors and socio-sexual interactions
Source: Front Neurosci. 2015 Apr 9;9:111. doi: 10.3389/fnins.2015.00111 (PMC4391029; doi:10.3389/fnins.2015.00111)
Supplement: Supplementary file 2 [file DataSheet2.PDF]

PLOS ONE

The "ram effect": a "non classical" mechanism for inducing LH surges in sheep

--Manuscript Draft--

**Manuscript Number:**	PONE-D-15-05481
**Article Type:**	Research Article
**Full Title:**	The "ram effect": a "non classical" mechanism for inducing LH surges in sheep
**Short Title:**	the LH surge and the "ram effect"
**Corresponding Author:**	claude fabre<br>CNRS<br>Nouzilly, FRANCE
**Keywords:**	sheep; LH; oestradiol; ram effect; spontaneous ovulation; induced ovulation; noradrenalin
**Abstract:**	During spring sheep do not ovulate but exposure to a ram can induce ovulation. In some ewes the LH surge is induced immediately after exposure to a ram raising questions about the control of this surge. Our first aim was to determine the plasma concentrations of oestradiol (E2) in anoestrous Ile de France (experiments 1 and 2) and Mérinos d'Arles (experiment 1) ewes before and after the "ram effect" in ewes that had a "precocious" LH surge (experiments 1 and 2), a "normal" surge (experiments 1 and 2) or in ewes that had not had a surge by 56h ("late" exp 1). E2 significantly varied with time but was not different at the time of the ram introduction among ewes with "precocious" "normal" or "late" surges and "precocious" LH surges are not preceded by a large increase in E2 contrary to "normal" surges. These results show that in "precocious" ewes the LH surge was not induced by a classic E2 positive feedback. Our second aim was to test whether noradrenalin (NA) could be implicated in the LH response to the "ram effect". Using double labelling for Fos and tyrosine hydroxylase (TH) we showed that exposure of anoestrous ewes to a ram induced a higher density of cells positive for both Fos and TH in A1 nucleus and Locus Coeruleus complex than the control situation. Administration by retrodialysis in the preoptic area, of noradrenalin increased the proportion of ewe responding to ram odor whereas treatment with α1 antagonist Prazosin decreased the LH pulse frequency and amplitude induced by a sexually active ram. These results suggested that NA could be partly responsible for the male induced LH secretion as observed in induced ovulators.
**Order of Authors:**	claude fabre
	Audrey Chanvallon
	Joelle Dupont
	Lionel Lardic
	Didier Lomet
	Stephanie Martinet
	Rex Scaramuzzi
**Opposed Reviewers:**	IJ Clarke<br>monash university<br><br>became suddently interested in the ram effect so is a direct competitor
	JT Smith<br>University of Western Australia<br><br>became recently interested in the ram effect, direct competitor
**Additional Information:**	
**Question**	**Response**
**Financial Disclosure**	The research was supported by Grants from the Région Centre (Durarep N° 2008 00030333 & Durarep 2 N°2011 00064290) and the European Union Framework 6

Powered by Editorial Manager® and ProduXion Manager® from Aries Systems Corporation

Please describe all sources of funding that have supported your work. A complete funding statement should do the following:<br><br>Include **grant numbers and the URLs** of any funder's website. Use the full name, not acronyms, of funding institutions, and use initials to identify authors who received the funding.<br>**Describe the role** of any sponsors or funders in the study design, data collection and analysis, decision to publish, or preparation of the manuscript. If they had no role in any of the above, include this sentence at the end of your statement: "*The funders had no role in study design, data collection and analysis, decision to publish, or preparation of the manuscript.*"<br><br>If the study was **unfunded**, provide a statement that clearly indicates this, for example: "*The author(s) received no specific funding for this work.*"<br><br>* typeset	funding program (MEXC-CT-2006-042499). RJS was the recipient of an EU Marie Curie Chair of Excellence (MEXC-CT-2006-042499). A C was recipient of a PhD grant funded by the “Convention Industrielle de Formation par la Recherche” between the “Centre Interrégional d’Information et de Recherche en Production Ovine” and the “Association Nationale de la Recherche et de la Technologie”.
**Competing Interests**<br><br>You are responsible for recognizing and disclosing on behalf of all authors any competing interest that could be perceived to bias their work, acknowledging all financial support and any other relevant financial or non-financial competing interests.<br><br>Do any authors of this manuscript have competing interests (as described in the PLOS Policy on Declaration and Evaluation of Competing Interests)?<br><br>**If yes**, please provide details about any and all competing interests in the box below. Your response should begin with this statement: *I have read the journal's policy and the authors of this manuscript have the following competing interests:*<br><br>**If no** authors have any competing interests to declare, please enter this statement in the box: "*The authors have*	The authors have declared that no competing interests exist."

declared that no competing interests exist."<br><br>* typeset	
**Ethics Statement**<br><br>You must provide an ethics statement if your study involved human participants, specimens or tissue samples, or vertebrate animals, embryos or tissues. All information entered here should **also be included in the Methods section** of your manuscript. Please write "N/A" if your study does not require an ethics statement.<br><br>**Human Subject Research (involved human participants and/or tissue)**<br><br>All research involving human participants must have been approved by the authors' Institutional Review Board (IRB) or an equivalent committee, and all clinical investigation must have been conducted according to the principles expressed in the Declaration of Helsinki. Informed consent, written or oral, should also have been obtained from the participants. If no consent was given, the reason must be explained (e.g. the data were analyzed anonymously) and reported. The form of consent (written/oral), or reason for lack of consent, should be indicated in the Methods section of your manuscript.<br><br>Please enter the name of the IRB or Ethics Committee that approved this study in the space below. Include the approval number and/or a statement indicating approval of this research.<br><br>**Animal Research (involved vertebrate animals, embryos or tissues)**<br><br>All animal work must have been conducted according to relevant national and international guidelines. If your study involved non-human primates, you must provide details regarding animal welfare and steps taken to ameliorate suffering; this is in accordance with the recommendations of the Weatherall report, "The use of non-human primates in research." The relevant guidelines followed and the committee that approved	The experiment was carried out in accordance with French and European regulations on the care and welfare of animals in research and with the authorization of the French Ministry of Agriculture (permit N° 006259) and the approval of the local ethics committee (permit N° 2012-01-2, comité d'éthique en expérimentation animale Val de Loire", N°19).

the study should be identified in the ethics statement.<br><br>If anesthesia, euthanasia or any kind of animal sacrifice is part of the study, please include briefly in your statement which substances and/or methods were applied.<br><br>Please enter the name of your Institutional Animal Care and Use Committee (IACUC) or other relevant ethics board, and indicate whether they approved this research or granted a formal waiver of ethical approval. Also include an approval number if one was obtained.<br><br>**Field Permit**<br><br>Please indicate the name of the institution or the relevant body that granted permission.	
**Data Availability**<br><br>PLOS journals require authors to make all data underlying the findings described in their manuscript fully available, without restriction and from the time of publication, with only rare exceptions to address legal and ethical concerns (see the PLOS Data Policy and FAQ for further details). When submitting a manuscript, authors must provide a Data Availability Statement that describes where the data underlying their manuscript can be found.<br><br>Your answers to the following constitute your statement about data availability and will be included with the article in the event of publication. **Please note that simply stating 'data available on request from the author' is not acceptable.** ***If, however, your data are only available upon request from the author(s), you must answer "No" to the first question below, and explain your exceptional situation in the text box provided.***<br><br>Do the authors confirm that all data underlying the findings described in their manuscript are fully available without restriction?	Yes - all data are fully available without restriction
Please describe where your data may be found, writing in full sentences. **Your answers should be entered into the box below and will be published in the form you provide them, if your manuscript is**	All relevant data are within the paper

**accepted.** If you are copying our sample text below, please ensure you replace any instances of XXX with the appropriate details.<br><br>If your data are all contained within the paper and/or Supporting Information files, please state this in your answer below. For example, "All relevant data are within the paper and its Supporting Information files."<br>If your data are held or will be held in a public repository, include URLs, accession numbers or DOIs. For example, "All XXX files are available from the XXX database (accession number(s) XXX, XXX)." If this information will only be available after acceptance, please indicate this by ticking the box below.<br>If neither of these applies but you are able to provide details of access elsewhere, with or without limitations, please do so in the box below. For example:<br><br>"Data are available from the XXX Institutional Data Access / Ethics Committee for researchers who meet the criteria for access to confidential data."<br><br>"Data are from the XXX study whose authors may be contacted at XXX."<br><br>* typeset	
Additional data availability information:	

Sheep are seasonal, spontaneous ovulators. During spring and early summer, when females are sexually quiescent, they do not ovulate but exposure to a ram can induce ovulation. In some females the LH surge is induced immediately after exposure ("precocious") to the ram raising questions about the control of this LH surge. The first aim of this study was to understand the role of oestradiol . To achieve this we measured plasma concentrations of oestradiol of anoestrous ewes before and after the "ram effect" in ewes that had "precocious" LH surges and compare them with ewes that had LH surges later. Our second aim was to test whether noradrenalin (NA) could be implicated in the LH response to the "ram effect" by immunochemical detection of Fos in NA neurons and by pharmacological manipulation of the noradrenergic system.

Our results show for the first time, that in ewes with precocious LH surge that occurs immediately after the rams are introduced, the LH surge is not preceded by increased concentrations of oestradiol indicating that the mechanism involved is different from the classical oestradiol positive feedback. We also show that exposure of anoestrous ewes to a ram induced a higher density of cells positive for both Fos and tyrosine hydroxylase in A1 nucleus and Locus Coeruleus complex than the control situation and that we can modulate the LH response to the ram by manipulating the NA system . These results suggested that NA could be partly responsible for the male induced LH secretion as observed in induced ovulators

We are submitting this paper as a research article

Suggested editors:

Opposed reviewers:

IJ Clarke, JT Smith and any of their close collaborators

Manuscript
Click here to download Manuscript: art E2 NA plos9 mars.doc

The “ram effect”: a "non classical” mechanism for inducing LH surges in sheep

Claude Fabre-Nys [1*], Audrey Chanvallon [1, 2], Joëlle Dupont [1], Lionel Lardic [1], Didier Lomet [1], Stephanie Martinet[1], Rex J Scaramuzzi [3]

[1]UMR 7247 Physiologie de la Reproduction et des Comportements, CNRS, INRA, Université de Tours, Institut français du cheval et de l’équitation, 37380 Nouzilly, France

[2] Present address: Institut de l’élevage, –Oniris – BP 40706 – 44307 Nantes France

[3]Department of Comparative Biomedical Sciences, Royal Veterinary College, Hawkshead Lane South Mimms, Hertfordshire AL9 7TA, UK

*Corresponding author

E-mail : claude.fabre@tours.inra.fr (CFN)

Abstract 300 mots max actuellt 281

During spring sheep do not ovulate but exposure to a ram can induce ovulation. In some ewes the LH surge is induced immediately after exposure to a ram raising questions about the control of this surge. Our first aim was to determine the plasma concentrations of oestradiol (E2) in anoestrous Ile de France (experiments 1 and 2) and Mérinos d'Arles (experiment 1) ewes before and after the "ram effect" in ewes that had a "precocious" LH surge (experiments 1 and 2), a "normal" surge (experiments 1 and 2) or in ewes that had not had a surge by 56h ("late" exp 1). E2 significantly varied with time but was not different at the time of the ram introduction among ewes with "precocious" "normal" or "late" surges and "precocious" LH surges are not preceded by a large increase in E2 contrary to "normal" surges. These results show that in "precocious" ewes the LH surge was not induced by a classic E2 positive feedback. Our second aim was to test whether noradrenalin (NA) could be implicated in the LH response to the "ram effect". Using double labelling for Fos and tyrosine hydroxylase (TH) we showed that exposure of anoestrous ewes to a ram induced a higher density of cells positive for both Fos and TH in A1 nucleus and Locus Coeruleus complex than the control situation. Administration by retrodialysis in the preoptic area, of noradrenalin increased the proportion of ewe responding to ram odor whereas treatment with α1 antagonist Prazosin decreased the LH pulse frequency and amplitude induced by a sexually active ram. These results suggested that NA could be partly responsible for the male induced LH secretion as observed in induced ovulators.

Introduction

In all mammalian species that have been studied, ovulation is caused by the secretion from the adenohypophysis, of a large quantity of luteinizing hormone over a relatively short period: the LH surge. The LH surge is induced by an increase in secretion of the hypothalamic neuropeptide, gonadotrophin releasing hormone (GnRH). In species such as the sheep, the rat, the rhesus monkey and the human, the preovulatory secretions of GnRH and LH are tightly controlled by oestradiol secreted by dominant follicle(s). In these species, the circulating concentration of oestradiol is elevated for several hours before a LH surge is detected [1-5]. In ovariectomized ewes, rats and rhesus monkeys, the LH surge commences between 10 and 24 hours after exogenous oestradiol and independently of the mode of its administration: intramuscular [6], intravenous [7] or by sub-cutaneous implant [8-10].

In "induced ovulators", such as the rabbit, the cat and the ferret the increases in GnRH and LH are also preceded by a pre-ovulatory increase in the concentration of oestradiol [11-14] but this rise alone is not sufficient to induce normal LH surges and ovulations [15-17]. These surges are only induced if the females are mated [18] although in some species (e.g. mink) pairing with a male is a sufficient stimulus [19-20]. The noradrenergic system is considered to have a central role in this phenomenon. In the rabbit and ferret mating has been shown to activate noradrenergic neurons [21-22]; and the extracellular concentration of noradrenalin in the mediobasal hypothalamus increases rapidly just prior

to GnRH [23]. This increase can be reduced by an α1 antagonist administered in the arcuate nucleus [24]. Conversely, mating alone has a very limited effect on the induction of a LH surge without the sensitizing effect of oestradiol. For example mating alone induced an LH surge in only in 1 of 10 chronically ovariectomized does [25].

In sheep, reproduction is seasonal and ewes stop cycling as day length increases (anoestrus). During anoestrus their ovaries secrete very little oestradiol, there are no spontaneous pre-ovulatory LH surges and thus, the ewes do not ovulate. The introduction of a sexually active ram into a group of seasonally anoestrous ewes will induce an immediate increase in the pulsatile secretion of LH in close to 100% of ewes [26] and in a variable proportion of ewes initiates a sequence of physiological events that culminate in a LH surge and ovulation [26-28]. This socio-sexual stimulation is often referred to as the “ram effect”. In most ewes this male induced LH surge is preceded by a sustained increase in the plasma concentration of oestradiol lasting between 8 and 56 hours [29] similarly to what happens in cyclic ewes during the breeding season [3, 4].

However, there have been consistent reports that small numbers of anoestrous ewes stimulated by the “ram effect” have "precocious" LH surges, defined as a LH surge starting between 0 and 8 hours after the introduction of rams [30-31]. But, the causes of these early surges have not been studied. In our laboratory precocious LH surges were seen in about 15% Ile-de-France and Mérinos d’Arles ewes [26, 29]. This time interval is much shorter than that observed when the LH surge is induced with exogenous oestradiol [6, 9] and the LH surge occurs too soon to be explained by the normal oestradiol-induced positive feedback mechanism.

One explanation is that although these ewes were anoestrous, they were on the verge of ovulating and that the precocious LH surge was simply a spontaneous LH surge that had been induced by a normal oestradiol-induced positive feedback just before or at the time of the “ram effect”. If this is so, then the circulating concentrations of oestradiol in these ewes should already be elevated at the time of introduction of the rams. An alternative explanation is that in these animals, the LH surge was induced by the contact with the ram, by a mechanism different from the classic oestradiol-induced positive feedback mechanism and perhaps closer to the mechanism responsible for the LH surge in induced ovulators. In fact several authors have suggested that the “dualistic” concept of ovulation as either spontaneous or induced is an over simplification and that the neural circuitry underlying induced ovulation also exists in species that ovulate “spontaneously” [32-33]. Some authors even suggest that induced ovulation is the ancestral mode of pre-ovulatory LH secretion [34]. In sheep contact with a sexual partner is known to have profound effects on the timing of reproductive events at all stages of reproductive life; it hastens puberty [35], induces ovulation during seasonal anoestrus [28] or lactational anoestrus [36] and modifies the latency of the LH surge during the breeding season [40]. In one study

this effect was observed in oestradiol-treated ovariectomized ewes and is therefore the result of a direct stimulation of the hypothalamo-hypophyseal complex that does not involve ovarian feedback [38]. The pathway involved it not known but, increases in the extracellular concentrations of noradrenalin were detected in the posterior part of the preoptic area of ewes exposed to a sexually active ram and to a lesser extent, to his odor [39]. This supported the possibility of a role of noradrenalin in the male-induced LH secretion in anoestrous sheep.

The aim of this study was to determine the concentrations of oestradiol in jugular venous plasma of anoestrous ewes immediately before and after the "ram effect" and to compare these patterns of oestradiol secretion in ewes with precocious and normal LH surges in response to the "ram effect". In a second study we aimed to determine if noradrenergic neurons were activated during the "ram effect" and if by modulating this system, we could modify the response of ewes to the "ram effect" or to ram odor.

Material and Methods

The experiment was carried out in accordance with French and European regulations on the care and welfare of animals in research and with the authorization of the French Ministry of Agriculture (permit N° 006259) and the approval of the local ethics committee (permit N° 2012-01-2, comité d'éthique en expérimentation animale Val de Loire", N°19).

The Relationship between the ram-induced LH surge and oestradiol

Experiment 1

The plasma samples analyzed in experiment 1(the "precocious LH surge" study) were selected from a set of samples from another study the results of which have already been published [26]. In the published experiment we studied the pattern of plasma LH after the "ram effect" in Ile de France (IF) and Mérinos d'Arles (M) ewes some of which had "precocious LH surges". In the present study sets of samples from ewes with precocious LH surges ("precocious" n=6 for each breed) defined as one starting between 0 and 4 hours after the introduction of rams were compared to ewes with a normal LH surge defined as one starting between 16 and 28 hours after the introduction of rams ("normal" IF; n=7 and M; n=8). The characteristics of the LH surge in these two groups are presented table 1. A third group consisting of ewes that had no evidence of an LH surge starting before the end of the experiment at 56h ("late LH surge" IF; n=4 and M; n=1) was included. The concentration of oestradiol was determined in plasma samples collected 24, 22, 20, 1.5 and 0h before the introduction of rams and at 2, 4, and 8h after

the introduction of rams. Details of the blood sampling and processing procedures have been published [26].

Table 1. Characteristics of the LH surge in the animals selected for study.

	Groups	Number of animals	Latency of the onset of the LH surge (h)	Duration of the LH surge (h)	Maximum concentration of LH (ng/mL)
exp. 1	“precocious”	12	2.17± 0.47	14.83±0.62	33.25±3.98
	“normal”	15	17.87±1.16	15.07±1.22	28.50±1.57
	“late”	5	> 56		5.55±3.51
exp. 2	*year 1*				
	“precocious”	4	1.50±0.50	17.50±2.22	24.87±7.54
	“normal”	15	21.07±1.22	15.73±0.88	37.76±4.75
	year 2				
	“precocious”	4	1.06±0.36	18.94±1.86	35.68±2.36
	“normal”	11	19.27±0.67	18.91±1.86	36.13±3.03

Data are expressed as mean ± SEM. The latency of the onset of the surge was defined as the time of first observed concentration of LH that was more than 3 standard deviations above the baseline and preceding an increase in LH of at least 4 hours and with at least one value above 10ng/mL [9, 45]. The surge was considered to finish when the concentrations fell below 10% of the maximum concentration.

Experiment 2

A second experiment (the “follow-up” study) was designed *de novo* to confirm and extend the results of Experiment 1. The experiment used 30 mature anoestrous Ile de France ewes. They were subjected to the “ram effect” at the end of anoestrus (July). The experiment was replicated the following year with the 28 ewes that were still alive. The ewes were housed on straw bedding and were isolated from all contact with rams until the “ram effect”. They were fed a maintenance diet of hay supplemented with concentrate and with free access to water. Two days before the experiment a catheter was introduced in the jugular vein for blood sampling. Blood samples collected between 0 and 56h after the introduction of the rams were analyzed for LH. These data were then used to identify and select ewes with a “precocious” LH surge (n=4 each year, 2 of which were the same) or ewes with a “normal” LH (n=15 year 1 and n=11 year 2, 7 were selected on both years) as defined for experiment 1. Characteristics of the LH surges in these ewes are shown in table 1. The samples from these ewes were then analyzed for oestradiol as follows: Year 1; -21h and -19h before the introduction of rams and Year

2; -24h, -20h, -16h, -12h and then for both years every 2 hours between -6h and 8h relative to the introduction of rams.

The role of the noradrenergic system in the male-induced LH Surge

Experiment 3

In a third study (the "histological" study), noradrenergic neurons activated during the "ram effect" were identified using a double labelling procedure to detect presence of cFos a known marker of neuronal activation and tyrosine hydroxylase (TH) the rate limiting enzyme for synthesis of noradrenalin.

(a) Sample collection

During anoestrus, 11 ewes were habituated to human contact for two weeks before the experiment. They were divided in two random groups and treated either by the introduction of a sexually experienced adult ram into their pen (male-exposed group, n=6) or by continuing in isolation from rams (control group, n=5). 90 minutes after the introduction of the ram the ewes were killed by a licensed butcher following a protocol agreed by the ethics committee. The heads were then immediately perfused through both carotid arteries with 2L of 1% sodium nitrite in phosphate buffer (0.1 M, pH 7.6) and 4L of cold 4% paraformaldehyde (in phosphate buffer). The brains were then removed intact, post-fixed for 24h in 4% paraformaldehyde and then left in phosphate buffer containing 30% sucrose and 0.1% sodium azide. The fixed brains were cut transversely into three approximately equal blocks. Free floating sections of the posterior block (40µm) were cut on a freezing microtome (Leica, Paris, France) and stored in cryoprotectant (NaCl 9%, polyvinyl pyrrolidone 10%, saccharose 30%, ethylene glycol 30%, phosphate buffer 0.1 M pH 7.4) at 4°C. Every 10th section was stained with Cresyl violet to allow identification and delineation of noradrenergic brain areas according to Tillet and Thibault [40].

(b) Immunohistochemistry

For each animal, the noradrenergic nuclei were identified and sections selected to contain comparable structures among ewes (5 sections per ewe for A1, A2, A6, A7 and 3 for A5). The sections were first stained for Fos [43] using an affinity-purified rabbit polyclonal antibody raised against the Fos protein (Ab-2, PC38, Oncogene Research Products, Calbiochem, San Diego, CA, USA, diluted 1/60,000 in PBS-TA-BSA, 2 days, 4°C) and peroxidase-anti-peroxidase complex solution (Jackson Immunoresearch, West Grove, PA, USA, diluted in 1/1,000 in PBS-BSA, 4°C) visualized by 3-3'diaminobenzidine tetrahydrochloride (DAB, Sigma Chemical, St Louis, MO, USA) intensified with 0.3% nickel ammonium sulphate. The sections were then rinsed in phosphate-buffered saline (PBS, 10% phosphate buffer 0.1 M, pH 7.4, 0.9% NaCl in distilled water) and stained for TH [41] using a mouse

monoclonal antibody raised against TH (Chemicon International) followed by diluted 1/1000 and a peroxidase-antiperoxidase conjugated to sheep anti-mouse antibody (Jackson Immunoresearch) diluted 1/500 visualized 3-3'diaminobenzidine tetrahydrochloride (Sigma Chemical, St Louis, MO, USA).

(c) Quantification of immuno-labelling

Quantification of Fos positive cells (Fos-IR) was performed using an image analysis system equipped with software to analyze cell-count data (Mercator, Explora Nova, La Rochelle, France). To count Fos positive cells a microscope with a motorized stage and a video camera was connected to a computer with a color monitor. Using the program we established parameters of size, shape and threshold for grey scale to characterize Fos-IR positive cells [42]. The average background grey scale was automatically estimated for every section and subtracted from the original image before the software identified automatically the cells meeting the established parameters as Fos-IR positive cells. Each section was also examined visually and any suspect objects (ex dust) erased manually. For counting TH-IR and Fos-IR/TH-IR positive cells, a manual system (Biocom, Paris, France) was used. Images were viewed on a monitor by an observer who marked every immunoreactive cell on the screen. Because A6 and A7 overlap they were counted together as LC-A6.

Experiment 4

In a fourth study (the "pharmacological" study) we determined if we could enhance the short-term LH response to a sub-stimulating ram cue made of a handful of ram fleece by the local infusion of noradrenalin (experiment 4a) or block the short-term LH response to exposure to a sexually active ram by local administration of the noradrenergic α1 adrenergic antagonist Prazosin (experiment 4b).

Two to 4 weeks before the start of the experiment, 15 mature Ile-de-France ewes were fitted bilaterally, with guide cannulae directed at the posterior preoptic area using a technique which combined a stereotaxic method with lateral and frontal radiography [43]. The procedures were carried out under general anesthesia induced by the intravenous injection of thiopental (1g; Nesdonal, Specia Rhone Poulenc, Paris, France) and atropine sulfate (20mg; Lavoisier, Paris, France) and maintained by closed-circuit halothane (Bélamont, Neuilly, France). Full aseptic precautions were taken throughout. After surgery, ewes were injected with 5ml of Dexamethasone (Diurizone, Vetoquinol, Lure, France) daily for 3 days. The ewes were allowed a minimum of 2 weeks recovery time and during their recovery the ewes were habituated to handling and the presence of humans.

The day before the experiment, noradrenalin (Research Biochemical international N-112 Natick USA, 100ng/mL) was dissolved in 1 mL $HClO_4$ 0.25M. On the day of the experiment noradrenalin,

Prazosin (Sigma P7791 Saint Quentin Fallavier France, 100µg/mL) or their solvent (ringers solution pH6.5) were infused by retrodialysis at a rate of 2µl/min using microdialysis probes (Mab 6 Microbiotech Sweden) inserted into the guide cannulae. Infusion started half an hour before exposure to a handful of shorn fleece of rams that only induces some LH increase in part of the ewes (experiment 4a) or a sexually active ramthat induces a large and immediate increase in LH in all ewes (experiment 4b). Ewes acted as their own controls and the order of infusion of the treatment solution and the ringer control solution were random. Plasma samples were collected every 15 min for 3 hours before and 3 hours after exposure to the shorn fleece of rams (experiment 4a) or a ram (experiment 4b).

The concentrations of progesterone in plasma were measured once a week during the whole of experiment 4 and infusions were only done on ewes that had progesterone concentration <1ng/mL for at least 2 weeks. In both experiments 11 anoestrous ewes were available, 7 ewes were used in both experiments and 4 ewes were used in only 1 experiment.

At the end of experiment 4b all the females were killed. Free-floating frontal sections (40µm thick) were cut and stained with cresyl violet to facilitate histological identification of probes location as described for experiment 3.

Assay of Oestradiol

The concentrations of oestradiol in jugular venous plasma were determined using the HRP-oestradiol DIASource immunoassay ELISA kit (E2-EASIA / KAP0621; DIASource immunoassay SA, Louvain la Neuve, Belgium), adapted for the detection of oestradiol in ovine plasma [29]. The sensitivity of the assay is 0.39 pg/mL and its detection limit 0.78 pg/mL. The intra-assay and inter assay coefficients of variation were: 16.6% and 15.3% (at 0.46pg/mL), 11.9% and 11.7% (at 0.80pg/mL) and 4.6% and 4.7% (at 5.13pg/mL).

Assay of LH

The concentrations of LH in jugular venous plasma were determined using a radioimmunoassay [44]. The assay sensitivity was 0.16 ng/mL standard 1051-CY-LH (equivalent to 0.31 ng/mL NIH LH-S1). The intra-assay and inter assay coefficients of variation were: 4.4% and 10.3% respectively.
The onset of the LH surge was defined as the time of the first observed concentration of LH that was more than 3 standard deviations above the baseline and preceding an increase in LH of at least 4 hours and with at least one value above 10ng/mL [9, 45]. LH pulses were identified as previously described [26]. An animal was classified as having a short-term LH response if the number of LH pulses during the

3 hours after stimulation was superior to the number of pulses during the 3 hours before stimulation. The amplitude of a LH pulse was calculated as the difference between the maximum concentration of LH in the pulse and the concentration of LH in the sample before the start of the pulse [46].

Statistical analyses

Statistical tests were carried out using Statistica version 10 (Statsoft Inc.). The data involving oestradiol measured over time were analyzed using a mixed model ANOVA run under the general linear model with time as a repeated measure. Data from each year in experiment 2 were analyzed separately. Paired comparisons within treatments were carried when appropriate, using the Bonferroni correction.

The mean density of immunoreactive cells was calculated for each region of each animal and the overall median and inter-quartile values calculated for the two groups. Because the mean densities were not normally distributed, statistical comparisons were carried out using nonparametric tests: The Kruskal and Wallis test followed by the Mann Whitney U test to compare groups or the Friedman tests to compare the density among nuclei in each group.

The proportions of ewes showing increased pulsatile LH activity were compared by χ^2 tests. The LH pulse frequency and the amplitudes of LH pulses before and after stimulation and during the treated versus control session were compared using Wilcoxon test.
Differences were taken as statistically significant at $p < 0.05$ and as a trend as a p value between 0.05 and 0.10.

Results

The “precocious” surge study

The duration and amplitude of the LH surge did not differ between "precocious" and "normal" surges (Table 1) but concentrations of oestradiol in ewes with "precocious" "normal" or "late" LH surges did as illustrated in Fig. 1. They showed a significant effect of time ($p < 0.0001$) and of the type of response ($p = 0.029$), but no effect of breed ($p = 0.890$). An interaction was seen between breed and type of response ($p = 0.040$) and there was a trend for the interaction between type of response and time ($p = 0.064$). Paired comparisons showed that the concentrations of oestradiol in the samples taken at 2h, 4h and 8h after the introduction of the introduction rams all differed significantly from of those taken before the introduction of rams (all comparisons $p < 0.001$). Before the introduction of rams, there were no significant differences in the mean concentration of oestradiol among the types of response

("precocious”, 1.15 ± 0.15 pg/mL; "normal", 0.82 ± 0.08 pg/mL and "late" 1.06 ± 0.31 pg/mL). Following the introduction of rams the concentrations of oestradiol at 2h and 4h, in the "precocious" group (2h: 4.26 ± 0.27 pg/mL and 4h: 5.03 ± 0.65 pg/mL) were significantly different from those in "normal" (2h: 2.60 ± 0.27 pg/mL, $p < 0.005$ and 4h: 2.65 ± 0.32 pg/mL, $p < 0.001$) and significantly different at 4 h from the "late" responders (1.17 ± 0.36 pg/mL, $p < 0.002$).

Fig. 1. Changes in oestradiol concentrations in anoestrous Ile de France and Mérinos d’Arles ewes who presented LH surges at different time after ram introduction ("precocious" surge study). "precocious": ewes presenting a LH surge within 4 hours after male introduction; "normal": ewes presenting a LH surge between 16 and 28 hours after the introduction of rams; "late": ewes who did not present a surge before 56h. Data are expressed as Mean ± SEM.

The “follow-up” study

The concentrations of oestradiol in ewes with "precocious" LH surges compared to ewes with “normal” LH surges are illustrated in Fig. 2. In both years, the concentrations of oestradiol at the time of ram introduction (time 0) were not significantly different in ewes with "precocious" and “normal” LH surges and increased after the introduction of the ram.

Fig. 2. Changes in oestradiol concentrations before and after ram introduction in anoestrous Ile de France who presented LH surges at different time after ram introduction (follow up study). "precocious": ewes who presented a LH surge within 4 hours after male introduction; "normal": ewes presented a LH surge between 16 and 28 hours after the introduction of rams. A year 1 B year 2. Data are expressed as Mean ± SEM. * $p<0.03$, ** $p<0.01$ compared to “normal” at the same time.

The global analysis showed a significant effect of time for each year ($p<0.0001$) and an interaction between time and type of response (year 1 $p=0.002$, year 2 $p=0.0006$) but no significant effect of type of response ($p=0.21$ and 0.22 for years 1 and 2).

When the periods before and after the “ram effect” were analyzed separately, a significant effect of the type of response was observed. During year 1 type of response had no significant (P=0,28) effect before the "ram effect" (Mean oestradiol concentrations = 1.01±0.28 and 0.75±0.11pg/mL for “normal” and “precocious”). But type of response had a significant effect after the "ram effect" ($p<0.047$). Paired comparisons showed significant differences at 4 and 6 hours after the ram effect between the “precocious“ and “normal” groups (Fig. 2. ; +4h: 5.1±0.87 versus 2.43±0.32 pg/mL, $p<0.006$ and +6h: 5.66±1.25 versus 3.24±0.39 pg/mL $p<0.027$).

In the second year a significant effect of the type of response was observed before the “ram effect” ($p<0.03$) and paired comparison showed that oestradiol concentration at -16h before the “ram effect” was significantly greater in the “precocious” group (Fig. 2. ; 2.61±1.27 versus 0.82±0.16 pg/mL; $p<0.03$) and approached significance at -8h (3.02±0.81 versus 1.30±0.35 pg/mL; $p=0.056$). The mean concentrations of oestradiol after ram introduction were higher than before but, did not vary between type of response (“precocious”: 2.20±0.10, “normal”: 2.49±0.37 pg/mL).

The “histological” study

The fos immunoreactive cells were characterized by a dense black precipitate in their nuclei and the TH immunoreactive neurons were characterized by a brown precipitate in the cytoplasm so double labelled cells could be identified by the presence of a dark nucleus in a brown cytoplasm (Fig. 3).

Fig. 3. Photography illustrating neurons expressing Fos and TH proteins in a female exposed to a ram. arrows with dotted line: Fos alone ; arrows with filled line: Fos protein in a TH neuron ; star TH without Fos protein.

The density of Fos immunoreactive cells was significantly different between monoaminergic nuclei ($p<0.001$ in both groups) but did not differ between the females exposed to a control situation and to a ram (Fig. 4a). By contrast the proportion of TH immunoreactive cells that were also immunoreactive for Fos differed between groups and the proportion of double-labelled cells in A1ventrolateral medulla nucleus ($p<0.03$) and the locus coeruleus complex ($p<0.004$) was higher in ewes exposed to a ram than in the controls (Fig. 4b).

Fig. 4. Box Plot representations of the density of Fos-IR neurons (A) and proportion of TH-IR cells also containing Fos-IR (B) in the different noradrenergic nuclei in adult Ile de France ewes exposed to a ram or to a control situation. The bottom and top of the boxes are the first and third quartiles, the band inside the boxes is the median and the end of the whiskers are the minimum and maximum of all the data; * $p<0.03$, ** $p<0.004$ compared to the control group.

The “pharmacological” study

As shown in Fig. 5, the ends guide cannulae were all located in the posterior preoptic area (Anteroposterior coordinates= 32 from the Richard’s atlas [47]).

Fig. 5. Schematic representation of the localization of the tip of dialysis probes used in the “pharmacological study”. Numbers correspond to the different animals.

The infusion of noradrenalin into this region increased the proportion of ewes responding to ram odor (9/11 versus 5/11; p=0.03). The frequency of LH pulses after exposure to ram odor was higher than before in ewes infused with noradrenalin (Figure 6a; p<0.003) whereas exposure to ram odor for 3 hours had no significant effect in control ewes. Noradrenalin treatment did not affect the amplitude of LH pulses after exposure to ram odor (p=0.12; Fig. 6b).

Fig. 6. Box Plot representations of the effect of infusion with norepinephrine (100 ng/mL) on LH secretion induced in anoestrous Ile de France ewes by exposure to ram fleece. A pulse frequency, B pulse amplitude. ** Different from before the stimulation p<0.003.

Prazosin did not affect, the proportion of ewes responding to the “ram effect” (7/11 versus 8/11) and introduction of a ram increased LH pulse frequency in both control (p<0.003) and Prazosin-treated ewes (p<0.02; Fig. 7a). However, the frequency (p<0.03; Figures 7a) and the amplitude (p<0.04; Fig. 7b) of LH pulses following the “ram effect” were significantly lower after Prazosin treatment compared to the control treatment.

Fig. 7. **Box Plot representations of the effect of infusion with Prazosin (100 µg/mL) on LH secretion induced in anoestrous Ile de France ewes by exposure to a ram.** A: pulse frequency, B: pulse amplitude; * Different from before the stimulation p<0.02, ** different from before the stimulation p<0.003, diamond Prazosin different from ringer p<0.04.

Discussion

Mammals are traditionally considered as either spontaneous or induced ovulators. Sheep are spontaneous ovulators and the LH surge in cyclic ewes is driven by a large and sustained preovulatory rise of oestradiol [3, 5, 48]. During anoestrus the ewes’ ovaries are inactive and there are no spontaneous LH surges. However, anoestrous sheep can be induced to ovulate by the introduction of a ram. In the present study we showed for the first time that some ewes have LH surges occurring within 4 h of the introduction of rams ("precocious" LH surge) that are not preceded by large increased concentrations of oestradiol. We also showed that in anoestrous ewes, exposure to rams activated noradrenalin neuronal populations of the locus coeruleus complex and the A1 nucleus and that we can modify the LH response to male socio-sexual cues by locally manipulating the noradrenergic system in the preoptic area. Together these results suggest that the neural circuitry involved in the mechanism of the ram-induced

“precocious” LH surge in anoestrous ewes is different from the classic oestradiol positive feedback mechanism of induction of the LH surge. They also suggest that the noradrenergic system is involved in the LH response to male socio-sexual cues and extrapolating further, that “precocious” LH surges are a result of higher activation of the noradrenergic system possibly using a neuronal circuit partly similar to that in the mating-induced LH surge of induced ovulators.

In anoestrous ewes LH pulsatility is very low, ovaries secrete little oestradiol and no progesterone because ewes do no ovulate and do not have the corpora lutea. However introduction of a sexually active ram will induce an immediate increase in the pulsatile secretion of LH in close to 100% of ewes, and in a lower and variable proportion of ewes a LH surge [26]. In most ewes this LH surge occurs 12 to 56 hours after the introduction of a ram [26-28, 30] and is preceded by a sustained increase in the plasma concentration of oestradiol lasting between 8 and 56 hours with concentrations above 2.5 pg/mL 4 hours before the surge [29]. This is similar to what happens in cyclic ewes during the breeding season; when the LH surge is preceded by an increase in the plasma concentration of oestradiol that normally lasts more than 12 hours [3, 4]. The present study showed that this was not the case in ewes with a “precocious” LH surge although the durations and peak concentrations of these surges are not different from those occurring later. In our study these “precocious” LH surges were not preceded by a large increase in oestradiol concentrations and oestradiol concentrations at that time of ram introduction when the “precocious” surges were starting were still low. So these ewes were not on the verge of ovulating.

Another possibility is that, in these “precocious” ewes, LH surges were induced by small and short increases in oestradiol concentrations that we did not detect. Indeed, in the second year of experiment 2 when samples were collected over a longer interval some ewes had episodic increases in oestradiol secretion resulting in significantly higher concentration of oestradiol 16 hours before ram introduction compared to ewes with a normal LH surge. Studies on ovariectomized ewes treated with oestradiol implants during the breeding season have shown that LH surges can be induced by a much shorter period of elevated oestradiol than normally occurs in cycling ewes. A LH surge was observed in 1/12 Suffolk ewes exposed to oestradiol for 7 hours [49] and in 3/10 Ile de France ewes exposed to oestradiol for 3 hours [50]. However in these studies the reported concentrations of oestradiol were higher than in our study (8pg/mLversus 2.6 and 3pg/mL, 16h and 8h before the “ram effect”) and in the Ben Said et al study [50] the ewes were repeatedly exposed to rams to measure oestrus behavior. So it is very unlikely that the small increases in oestradiol observed in our study were alone, sufficient to induce “precocious” LH surges using the same mechanism as for the "normal" surges. We suggest that in our study and that of Ben Said et al [50] the LH surge was induced by a combination of oestradiol priming and male stimulation in a similar way as occurs in induced ovulators.

In induced ovulators an increase in oestradiol induces receptive behavior and allows mating and vaginal stimulation produced by mating, to induce the LH surge [18]. The noradrenergic system has a central role in this male induced LH surge [14, 18]. In rabbits and ferrets mating stimuli activate noradrenergic neurons in the locus coeruleus and the brainstem [21, 22]. This leads to a rapid increase in the extracellular concentrations of noradrenalin in the mediobasal hypothalamus just prior to the increase in GnRH [23] and this increase can be reduced by an α1 antagonist [24].

Anoestrous ewes are not sexually receptive and refuse all male courtship behavior so no vaginal stimulation can occur. But in a previous study we had shown that the extracellular concentrations of noradrenalin increased in the posterior preoptic area of anoestrous ewes exposed to a ram and to a lesser extent, ram odor [39]. Here we showed that as in induced ovulators, noradrenalin neurons from the locus coeruleus complex and from the brainstem were activated when anoestrous ewes were exposed to a sexual stimulus but in our study, without vaginal stimulation. The high sensitivity of ewes towards a sexual partner is not surprising because during the breeding season an increase in noradrenalin was observed in the mediobasal hypothalamus when estrus ewes were exposed to a picture of a ram's face and to a lesser extent to ram odor [51] whereas in oestrous rats noradrenalin increased only after vaginal stimulation [52]. Furthermore, in some induced ovulators such as the mink mating is not necessary and pairing alone is provides sufficient sexual stimulation to induce ovulation [19, 20].

In our study we have also been able to increase the effectiveness of male odor by the local administration of noradrenalin in the preoptic area of anoestrous ewes or to reduce the amplitude of the short-term LH response to the "ram effect" by an α1antagonist. These results suggest that in this context, noradrenalin has a facilitatory action on LH secretion in response to male cues. In sheep as in other species, noradrenergic neurons project fibers to the preoptic area [53, 54]. So these neurons are the probable origin of the increase in noradrenalin concentration in the preoptic area caused by exposure to a ram. Noradrenalin has been implicated in the control of LH secretion for several decades [55-60]. The mechanism by which noradrenalin modulates the activity of GnRH neurons remains unclear. Pharmacological manipulations of the noradrenergic system in spontaneous ovulators have complex and variable effects [57 - 60]. This has led authors to suggest that the role of noradrenalin on the onset of the LH surge in cyclic animals is permissive [56, 57] and not a trigger as in induced ovulators [14, 18, 61]. How the facilitating effect of noradrenalin we observed here is related to the onset of the LH surge is not clear. During the breeding season, the GnRH surge in ewes is the result of a high frequency of pulsatile events superimposed on a high but constant level of GnRH [47, 62]. We suggest that the facilitation of pulsatile LH secretion we observed here could ultimately stimulate the onset of "precocious" LH surge although but because of the low incidence of "precocious" responses and the inability to predict them it is impossible to test this hypothesis.

The existence of neural circuits underlying the male-induced LH surge in spontaneously ovulating species was first proposed almost 40 years ago [35, 63]. The most common examples cited to support this idea are the induction of an LH surge by mating in rats exposed to constant light [64, 65] and in hypogonadal mice grafted with GnRH neurons that only have a LH surge after mating [66]. But these are quite extreme experimental perturbations. Our study is the first to report that this phenomenon can occur under normal physiological conditions. Our data shows that in ewes, the neural circuitry required for both the oestradiol driven LH surge and the male induced LH surge can be activated under appropriate conditions. The activation of this neuronal system could also be responsible for the ability of a sexual partner to advance the time of the LH surge as has been reported for the sow [67], cow [68] and ewe [69] and even when the female has been ovariectomized and treated with exogenous oestradiol [38] and could also explain the LH surges observed in a some ovariectomized Ile de France after treatment with a very small amounts of oestradiol and repeated exposure to rams for the purposes of estrus detection [51]. Noradrenalin is implicated in both the neural circuits for spontaneous and induced ovulation. The questions now are why is one circuit activated in preference to the other and how are these two circuits connected?

Acknowledgments

We wish to thank the staff from INRA experimental stations in Nouzilly, Bourges and Frejorgues for the care of the animals and for their help with the experiments. We wish also to thank several MSc students, S Bertaux, C Buron, M David, A Dherbe, A Durand, J Gauthier, C Journault, A Pelletier and O Robineau, who participated in various aspects of these experiments.

References

1. Corker CS, Naftolin F, Exley D. Interrelationship between plasma luteinizing hormone and oestradiol in the human menstrual cycle. Nature 1969; 222:1063.
2. Smith MS, Freeman ME, Neill JD. The control of progesterone secretion during the estrous cycle and early pseudopregnancy in the rat: prolactin, gonadotropin and steroid levels associated with rescue of the corpus luteum of pseudopregnancy. Endocrinology 1975; 96: 219-226.

3. Hauger RL, Karsch FJ, Foster DL. A new concept for control of the estrous cycle of the ewe based on the temporal relationships between luteinizing hormone, estradiol and progesterone in peripheral serum and evidence that progesterone inhibits tonic LH secretion. Endocrinology. 1977; 101:807-817.
4. Karsch FJ, Weick RF, Butler WR, Dierschke DJ, Krey LC et al. Induced LH surges in the rhesus monkey: strength-duration characteristics of the estrogen stimulus. Endocrinology. 1973; 92: 1740-1747.
5. Karsch FJ, Foster DL, Legan SJ, Ryan KD, Peter GK. Control of the preovulatory endocrine events in the ewe: interrelationship of estradiol, progesterone, and luteinizing hormone. Endocrinology. 1979; 105: 421-426.
6. Scaramuzzi RJ, Tillson SA, Thorneycroft IH, Caldwell BV. Action of exogenous progesterone and estrogen on behavioral estrus and luteinizing hormone levels in the ovariectomized ewe. Endocrinology. 1971; 88: 1184-1189.
7. Banks JA, Mick C, Freeman ME. A possible cause for the differing responses of the luteinizing hormone surge mechanism of ovariectomized rats to short term exposure to estradiol. Endocrinology. 1980; 106: 1677-1681.
8. Goodman RL, Legan SJ, Ryan KD, Foster DL, Karsch FJ. Importance of variations in behavioural and feedback actions of oestradiol to the control of seasonal breeding in the ewe. J Endocrinol. 1981; 89: 229-240.
9. Caraty A, Locatelli A, Martin GB. Biphasic response in the secretion of gonadotrophin-releasing hormone in ovariectomized ewes injected with oestradiol. J Endocrinol. 1989; 123: 375-382.
10. Xia L, Van Vugt D, Alston EJ, Luckhaus J, Ferin M. A surge of gonadotropin-releasing hormone accompanies the estradiol-induced gonadotropin surge in the rhesus monkey. Endocrinology. 1992; 131: 2812-2820.
11.Wu CH, Blasco L, Flickinger GL, Mikhail G. Ovarian function in the preovulatory rabbit. Biol. Reprod. 1977; 17: 304-308.
12.Wildt DE, Chan SY, Seager SW, Chakraborty PK. Ovarian activity, circulating hormones, and sexual behavior in the cat. I. Relationships during the coitus-induced luteal phase and the estrous period without mating. Biol. Reprod. 1981; 25: 15-28.
13.Elias E, Bedrak E, Yagil R. Estradiol concentration in the serum of the one-humped camel (Camelus dromedarius) during the various reproductive stages. Gen. Comp. Endocrinol. 1984; 56: 258-264.
14. Ramirez VD, Soufi Wl. The neuroendocrine control of the rabbit ovarian cycle. In: Knobil E, Neill JD editors. The Physiology of Reproduction. 2nd ed., Vol. 2.New York: Raven Press; 1994. pp. 585-611.
15. Dufy-Barbe L, Dufy B, Vincent JD. Serum gonadotropin levels in the ovariectomized rabbit: effect of acute and chronic administration of estradiol. Biol Reprod. 1978; 18: 118-124.

16. Milligan SR. The feedback of exogenous steroids on LH release and ovulation in the intact female vole (Microtus agrestis). J Reprod Fertil. 1978; 54: 309-311.
17. Baum MJ, Carroll RS, Cherrv JA, Tobet SA. Steroidal control of behavioural, neuroendocrine and brain sexual differentiation: studies in a carnivore, the ferret J Neuroendocrinol. 1990; 2: 401-418.
18. Bakker J, Baum MJ. Neuroendocrine regulation of GnRH release in induced ovulators. Front Neuroendocrinol. 2000; 21: 220-262.
19. Adams CE. The reproductive status of female mink, *Mustela vision*, recorded as 'failed to mate'. J Reprod Fertil. 1973; 33: 527-529.
20. Adams CE. Observations on the induction of ovulation and expulsion of uterine eggs in the mink, *Mustela vison*. J Reprod Fertil. 1981; 63: 241-248.
21. Wersinger SR, Baum MJ. Sexually dimorphic activation of midbrain tyrosine hydroxylase neurons after mating or exposure to chemosensory cues in the ferret. Biol Reprod. 1997; 56:1407-1414.
22. Yang SP, Pau KY, Spies HG. Tyrosine hydroxylase and norepinephrine transporter mRNA levels increase in locus coeruleus after coitus in rabbits. J Mol Endocrinol. 1997; 19: 311-319.
23. Yang SP, Pau KYF, Hess DL, Spies HG. Sexual dimorphism in secretion of hypothalamic gonadotropin-releasing hormone and norepinephrine after coitus in rabbits. Endocrinology. 1996; 137: 2683-2693.
24. Yang SP, Pau KY, Airhart N, Spies HG. Attenuation of gonadotropin-releasing hormone reflex to coitus by alpha1-adrenergic receptor blockade in the rabbit. Proc. Soc. Exp. Biol. Med. 1998; 218: 204-209.
25. Goodman AL, Neill JD. Ovarian regulation of postcoital gonadotropin release in the rabbit: reexamination of a functional role for 20 alphadihydroprogesterone. Endocrinology. 1976; 99: 852-860.
26. Chanvallon A, Sagot L, Pottier E, Debus N, François D, et al. New insights into the influence of breed and time of the year on the response of ewes to the 'ram effect'. Animal 2011; 5: 1594-1604.
27. Martin GB, Oldham CM, Cognié Y and Pearce DL. The physiological responses of anovulatory ewes to the introduction of rams – a review. Livestock Production Science. 1986; 15: 219–247.
28. Ungerfeld R. Socio-sexual signalling and gonadal function: opportunities for reproductive management in domestic ruminants. Soc Reprod Fertil Suppl. 2007; 64: 207-221.
29. Fabre-Nys C, Chanvallon A, Debus N, François D, Bouvier F et al. Plasma and ovarian oestradiol and the variability in the LH surge induced in ewes by the ram effect. Reproduction. 2015 In Press
30. Oldham, CM, Martin, GB, Knight, TW. Stimulation of the seasonally anovular Merinos ewes by rams. I. Time from introduction of the rams to the preovulatory surge and ovulation. Anim Reprod Sci 1978; 1: 283–290.

31. Pearce, DT, Martin, GB, Oldham, CM. Corpora lutea with a short life-span induced by rams in seasonally anovulatory ewes are prevented by progesterone delaying the preovulatory surge of LH. J Reprod Fert. 1985; 75: 79–84.

32. Zarrow MX, Clark JH. Ovulation following vaginal stimulation in a spontaneous ovulator and its implications. J Endocrinol. 1968; 40: 343-352.

33. Jöchle W. Current research in coitus-induced ovulation: a review. J Reprod Fertil Suppl. 1975; 22: 165-207.

34. Conaway CH. Ecological adaptation and mammalian reproduction. Biol Reprod. 1971; 4: 239-247.

35. Dyrmundsson OR, Lees JL. Effect of rams on the onset breeding activity in the clun forest ewe lamb. J. agric. Sci. Camb. 1972; 79 : 269-271.

36. Mauleon P**,** Dauzier L. Variations de durée de l'anoestrus de lactation chez les brebis de race Ile-de-France. Ann. Biol. Anim. Bioch. Biophys. 1965 ; 5 : 131-143

37. Signoret JP. Effet de la présence du male sur les mécanismes de reproduction chez la femelle des mammifères. Reprod. Nutr. Dev. 1980 ; 20: 457-468

38. Signoret JP. Influence of the presence of rams on the luteinizing hormone surge after oestradiol benzoate injection in ovariectomized ewes. J Endocr. 1975; 64: 589-590.

39. Fabre-Nys C, Archer E, De la Riva C, Gelez H, Kendrick KM et al. Noradrenaline is also implicated in the “ram effect” Horm Behav. 2005; 48: 99.

40. Tillet Y, Thibault J. Catecholamine-containing neurons in the sheep brainstem and diencephalon, immunohistochemical study with tyrosine hydroxylase (TH) and dopamine-beta-hydroxylase (DBH) antibodies. J. Comp. Neurol. 1989; 290: 69-104.

41. Gelez H, Fabre-Nys C. Neural pathways involved in the endocrine response of anestrous ewes to the male or its odor. Neuroscience. 2006; 140, 791-800.

42. Chanvallon A, Fabre-Nys C. In sexually naive anestrous ewes, male odour is unable to induce a complete activation of olfactory systems. Behav. Brain Res. 2009; 205: 272-279.

43. Fabre-Nys C, Blache D, Lavenet C. A method for accurate implantation in the sheep brain. In Greenstein B editor. Neuroendocrine Research Methods, Implantation and Transfection Procedures. Harwood, Chur ; 1991. pp. 295–314

44. Montgomery GW, Martin GB, Pelletier J. Changes in pulsatile LH secretion after ovariectomy in Ile-de-France ewes in two seasons. J Reprod Fertil. 1985; 73*:* 173–183.

45. Moenter SM, Caraty A, Karsch FJ. The estradiol-induced surge of gonadotropin-releasing hormone in the ewe. Endocrinology. 1990; 127: 1375-1384.

46. Goodman RL, Karsch FJ. Pulsatile secretion of luteinizing hormone: differential suppression by ovarian steroids. Endocrinology. 1980; 107, 1286-1290.

47. Richard P. Atlas Stéréotaxique du Cerveau de Brebis. Paris: INRA. 1967.

48. Goodman RL. Neuroendocrine control of the ovine estrous cycle. In: Knobil E, Neill JD editors. The Physiology of Reproduction, 2nd Edition, Vol. 2. New York: Raven Press; 1994. pp. 659-709.

49. Evans NP, Dahl GE, Padmanabhan V, Thrun LA, Karsch FJ. Estradiol requirements for induction and maintenance of the gonadotropin-releasing hormone surge: implications for neuroendocrine processing of the estradiol signal. Endocrinology. 1997; 138: 5408-5414.

50. Ben Saïd S, Lomet D, Chesneau D, Lardic L, Canepa S, et al. Differential estradiol requirement for the induction of estrus behavior and the luteinizing hormone surge in two breeds of sheep. Biol Reprod. 2007; 76: 673-680.

51. Fabre-Nys C, Ohkura S, Kendrick KM. Male faces and odours evoke differential patterns of neurochemical release in the mediobasal hypothalamus of the ewe during oestrus: an insight into sexual motivation? Eur J Neurosci. 1997; 9: 1666-1677.

52. Etgen AM, Morales JC. Somatosensory stimuli evoke norepinephrine release in the anterior ventromedial hypothalamus of sexually receptive female rats. J. Neuroendocrinol. 2002; 14, 213-218.

53. Tillet Y, Batailler M, Thibault J. Neuronal projections to the medial preoptic area of the sheep, with special reference to monoaminergic afferents, immunohistochemical and retrograde tract tracing studies. J. Comp. Neurol. 1993; 330: 195-220.

54. Wright DE, Jennes L. Origin of noradrenergic projections to GnRH perikarya-containing areas in the medial septum-diagonal band and preoptic area. Brain Res. 1993; 621: 272-278.

55. Barraclough CA, Wise PM. The role of catecholamines in the regulation of pituitary luteinizing hormone and follicle-stimulating hormone secretion. Endocr Rev. 1982; 3: 91-119.

56. Herbison AE. Noradrenergic regulation of cyclic GnRH secretion. Rev Reprod. 1997; 2: 1-6.

57. Clarke IJ, Scott CJ, Pereira A, Pompolo S. The role of noradrenaline in the generation of the preovulatory LH surge in the ewe. Domest Anim Endocrinol. 2006; 30: 260-275.

58. Szawka RE, Poletini MO, Leite CM, Bernuci MP, Kalil B et al. Release of norepinephrine in the preoptic area activates anteroventral periventricular nucleus neurons and stimulates the surge of luteinizing hormone. Endocrinology. 2013; 154: 363-374.

59. Scott CJ, Cummins JT, Clarke IJ. Effects on plasma luteinizing hormone levels of microinjection of noradrenaline and adrenaline into the septo-preoptic area of the brain of the ovariectomized ewe: changes with season and chronic oestrogen treatment. J. Neuroendocr. 1992; 4: 131-141.

60. Goodman RL, Gibson M, Skinner DC, Lehman MN. Neuroendocrine control of pulsatile GnRH secretion during the ovarian cycle: evidence from the ewe. Reprod. Suppl. 2002; 59, 41-56

61. Spies HG, Pau KY, Yang SP. Coital and estrogen signals: a contrast in the preovulatory neuroendocrine networks of rabbits and rhesus monkeys. Biol Reprod. 1997; 56: 310-319.

62. Caraty A, Antoine C, Delaleu B, Locatelli A, Bouchard P, Gautron J.P et al. Nature and bioactivity of gonadotropin-releasing hormone (GnRH) secreted during the GnRH surge. Endocrinology. 1995; 136: 3452-3460.
63. Milligan SR. The feedback of exogenous steroids on LH release and ovulation in the intact female vole (Microtus agrestis). J. Reprod. Fertil. 1978; 54, 309-311
64. Hardy DF. The effect of constant light on the estrous cycle and behaviour of the female rat. Physiol Behav 1970; 5: 421-425.
65. Brown-Grant K, Davidson JM, Greig F. Induced ovulation in albino rats exposed to constant light. J Endocrinol 1973; 57: 7-22.
66. Gibson MJ, Wu TJ, Miller GM, Silverman AJ. What nature's knockout teaches us about GnRH activity, hypogonadal mice and neuronal grafts. Horm Behav. 1997; 31: 212-220.
67. Signoret JP, Du Mesnil du Buisson F, Mauléon P. Effect of mating on the onset and duration of ovulation in the sow. J Reprod Fert. 1972; 31: 327-330.
68. Randel RD, Short RE, Christensen DS, Bellows RA. Effects of various mating stimuli on the LH surge and ovulation time following synchronization of estrus in the bovine. J Anim Sci. 1973; 37:128-130.
69. Lindsay DR, Cognie Y, Pelletier J, Signoret JP. Influence of the presence of rams on the timing of ovulation and discharge of LH in ewes. Physiol Behav. 1975; 15: 423-426.

Figure
Click here to download high resolution image

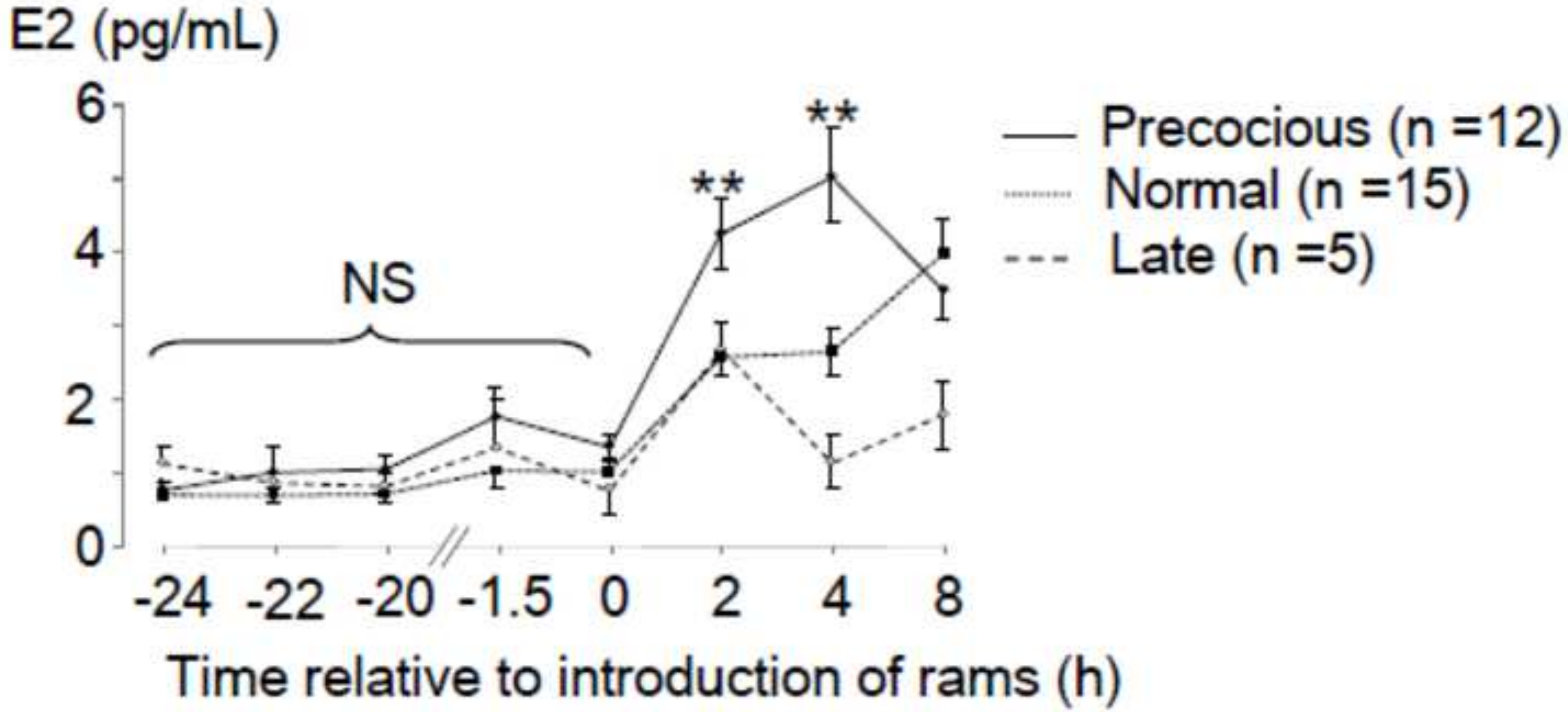

Figure
Click here to download high resolution image

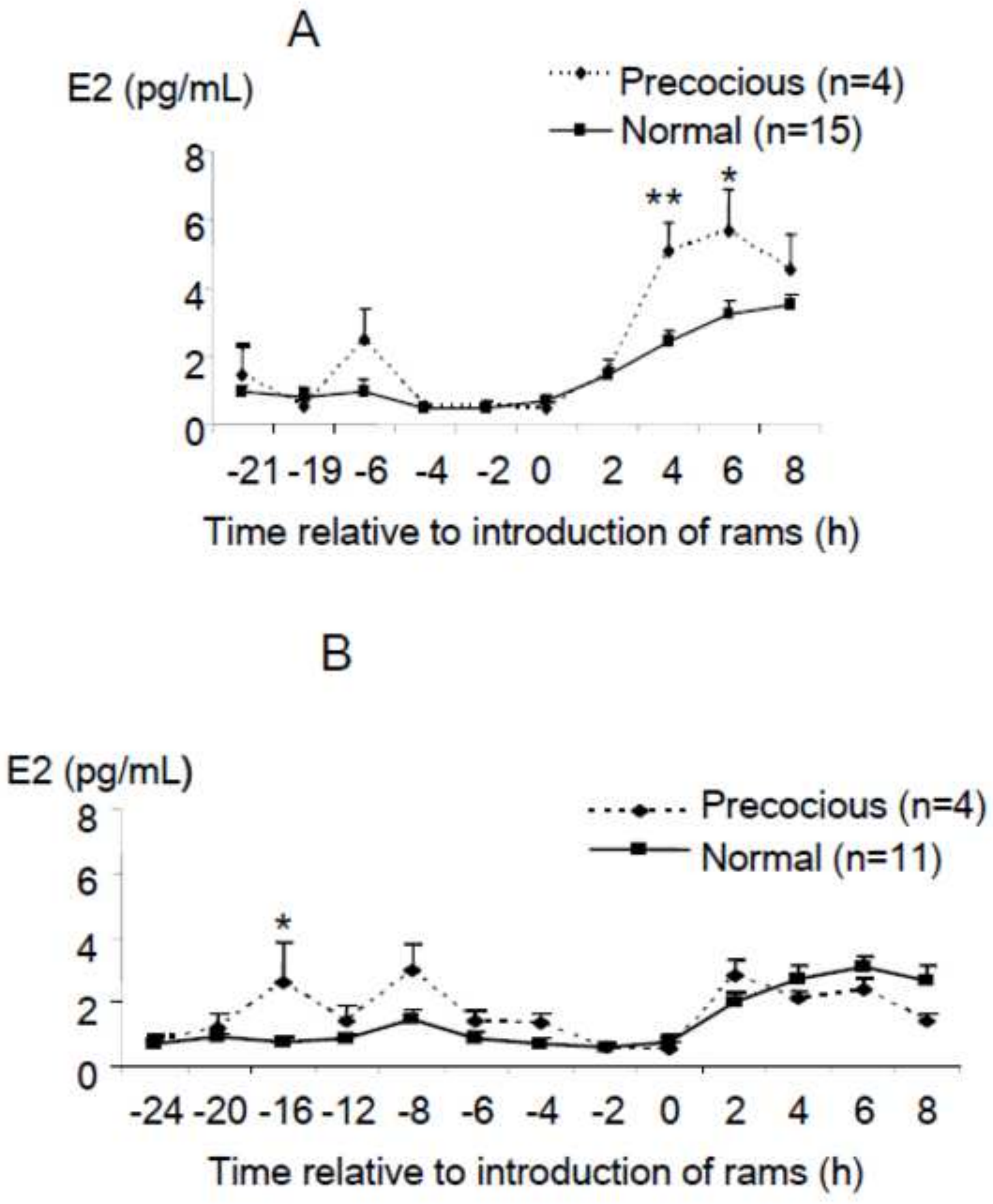

Figure
Click here to download high resolution image

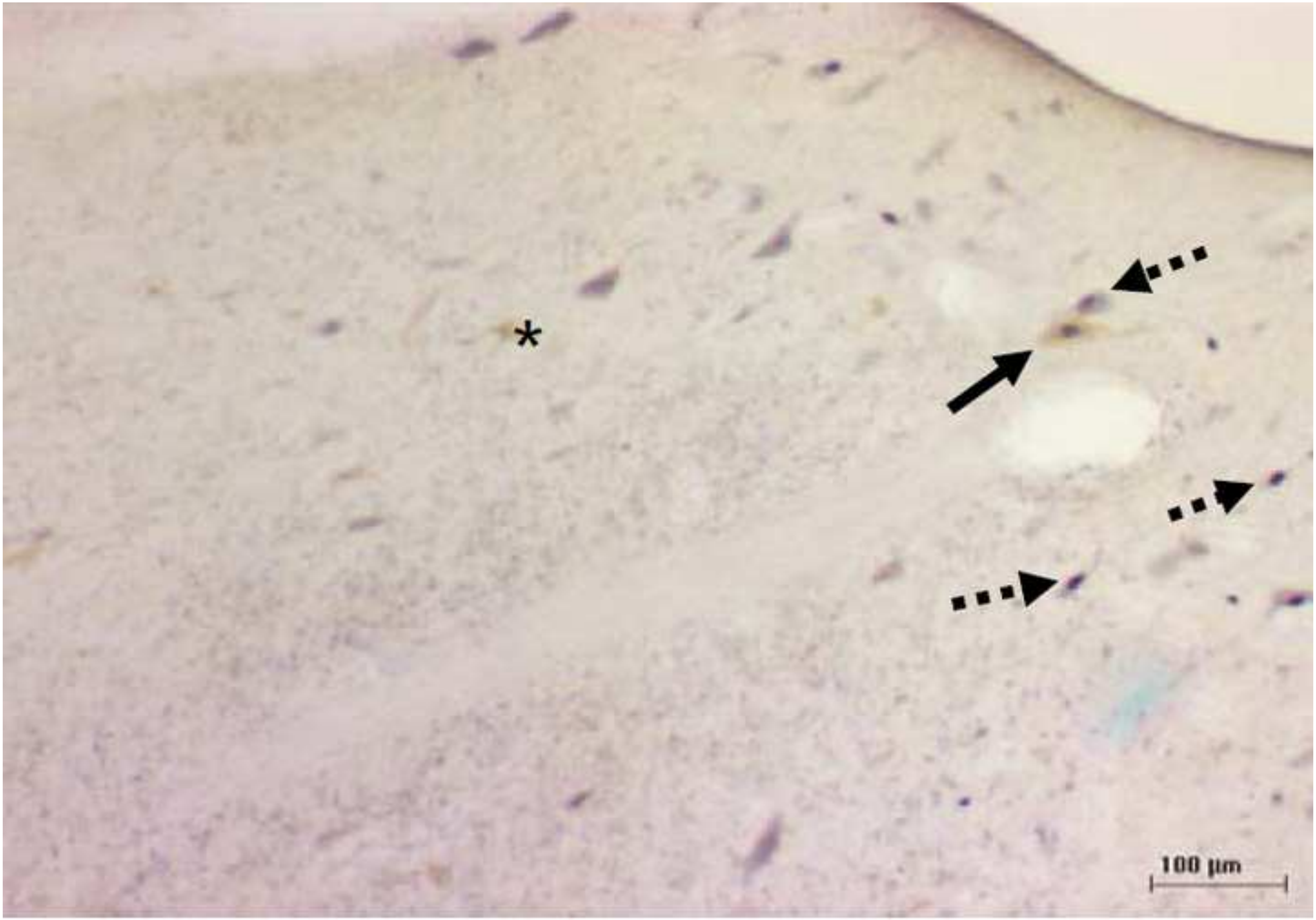

Figure
Click here to download high resolution image

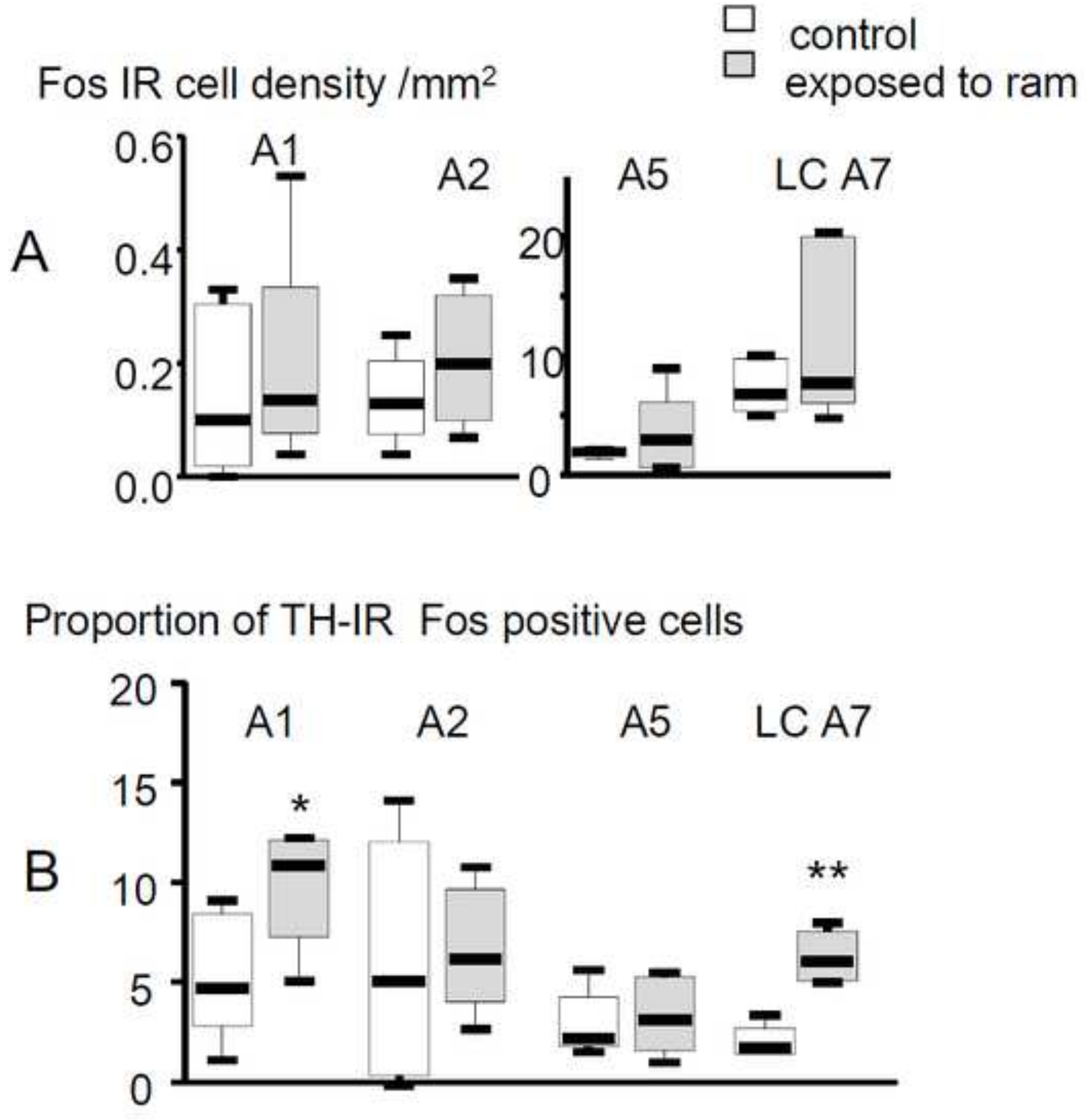

Figure
Click here to download high resolution image

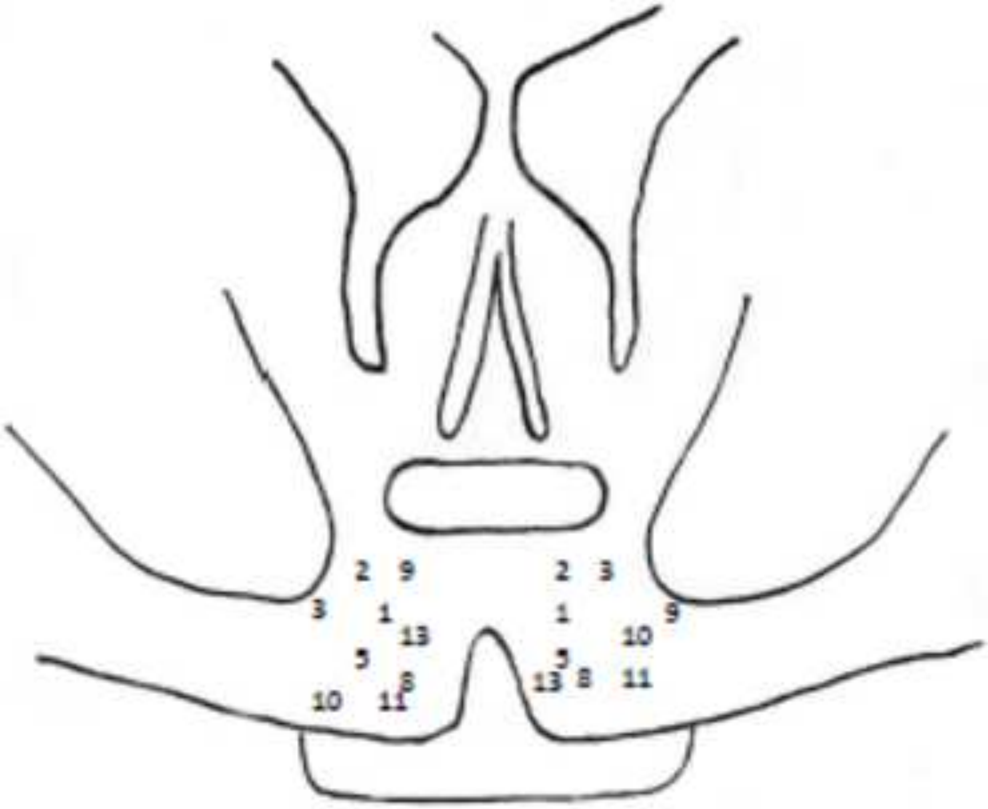

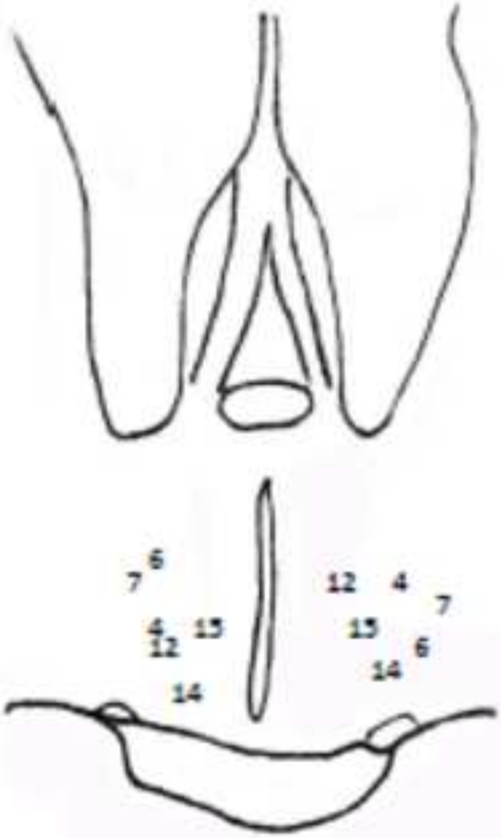

Figure
Click here to download high resolution image

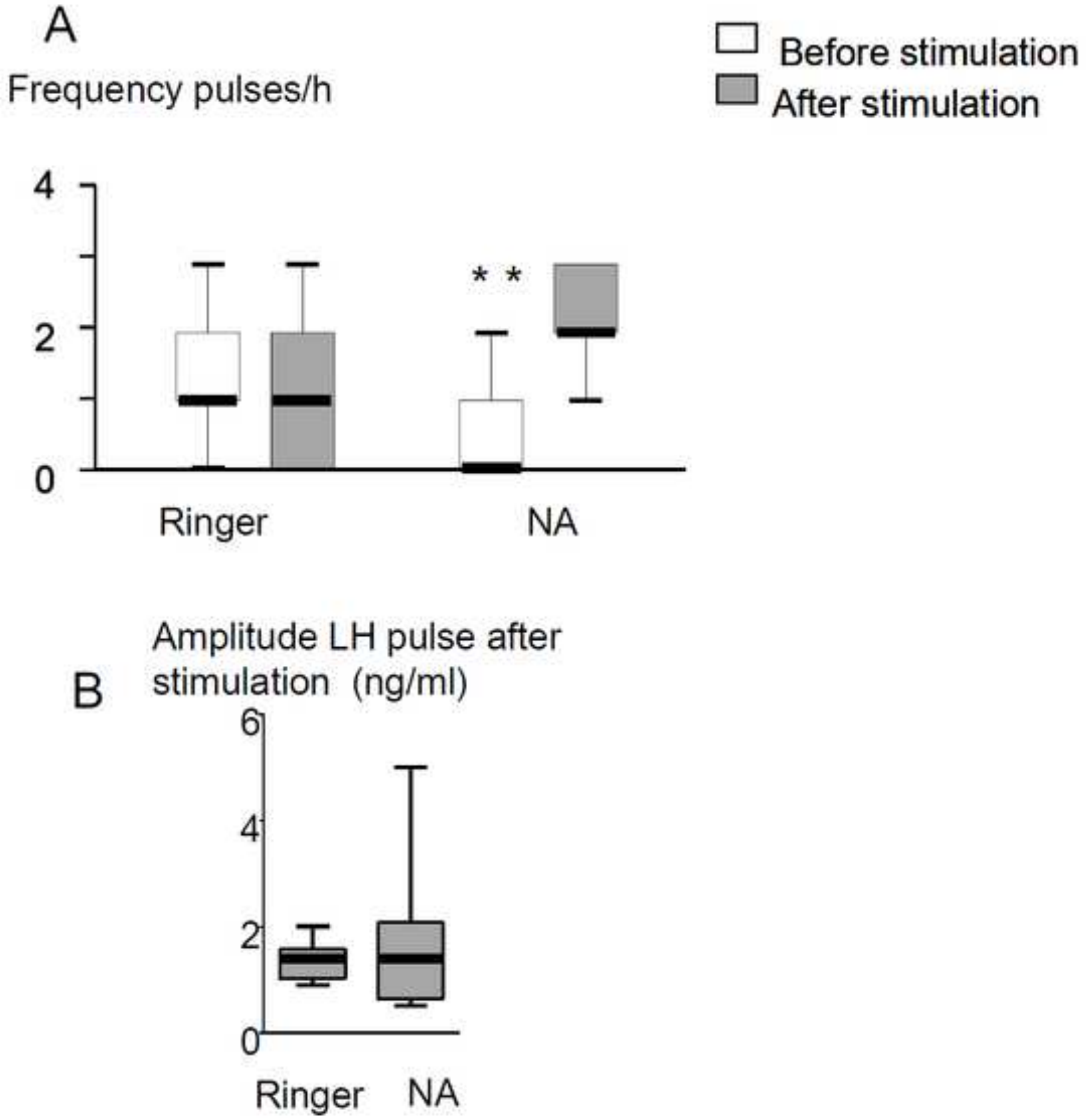

Figure
Click here to download high resolution image

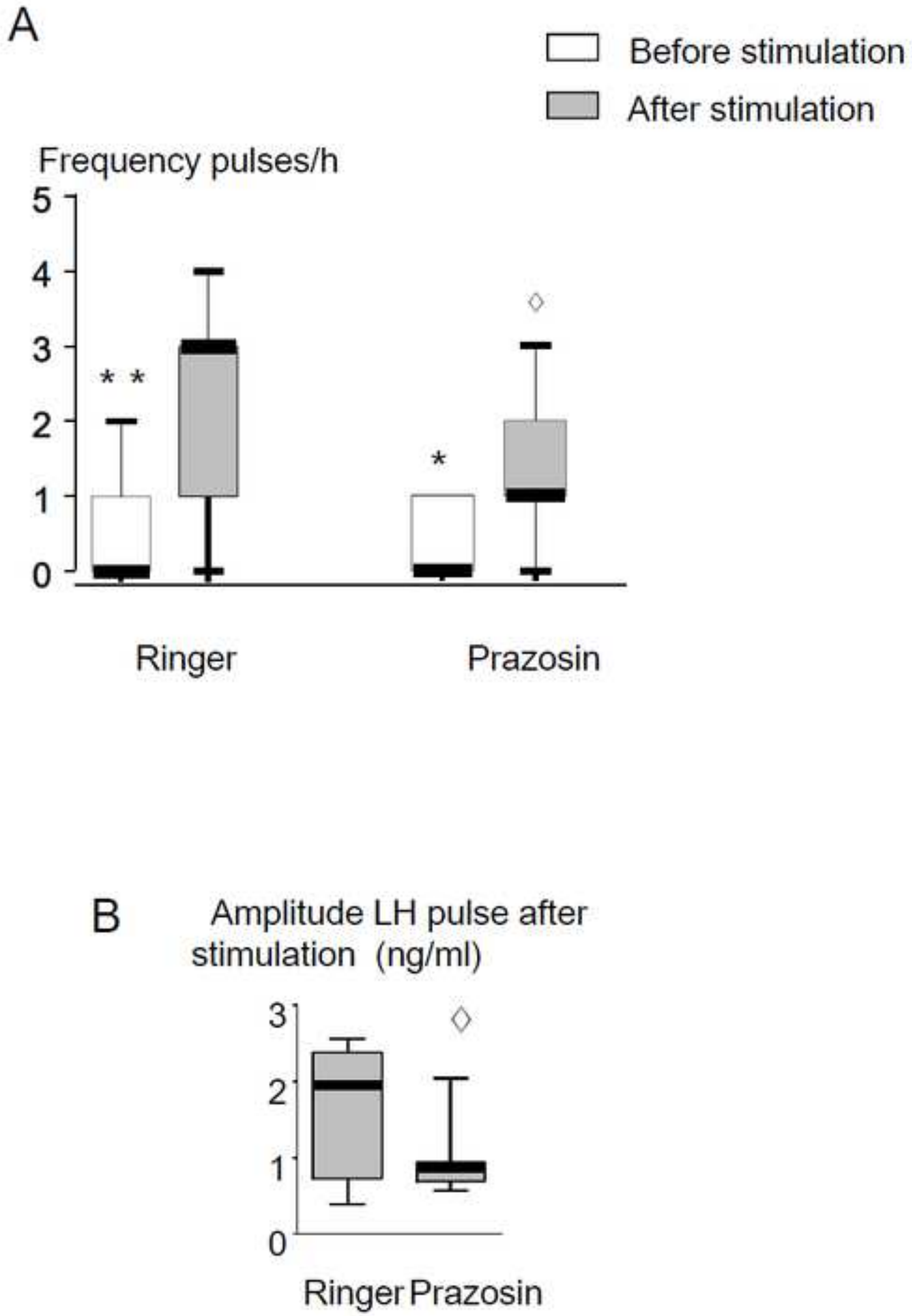

Supporting Information

Click here to download Supporting Information: reproduction proof.pdf

Supporting Information

Click here to download Supporting Information: NR-S-15-00160.pdf

Supporting Information
Click here to download Supporting Information: acceptation neuroreport.doc
